# Supplementary material for: Bis-Oxadiazole Assemblies as NO-Releasing Anticancer Agents
Source: Pharmaceutics. 2025 Nov 19;17(11):1494. doi: 10.3390/pharmaceutics17111494 (PMC12656327; doi:10.3390/pharmaceutics17111494)
Supplement: Supplementary file 1 [file pharmaceutics-17-01494-s001.zip › pharmaceutics-3952333-supplementary.pdf]

Bis-Oxadiazole Assemblies as NO-Releasing Anticancer Agents

Egor M. Matnurov [1,≠], Irina A. Stebletsova [2,3,≠], Alexander A. Larin [2], Jemma Arakelyan [1], Ivan V. Ananyev [4], Artem L. Gushchin [5], Leonid L. Fershtat[2,*], Maria V. Babak[1,*]

[1] Drug Discovery Lab, Department of Chemistry, City University of Hong Kong, 83 Tat Chee Avenue, Hong Kong SAR, China

[2] N.D. Zelinsky Institute of Organic Chemistry, Russian Academy of Sciences, 47 Leninsky Prosp., 119991 Moscow, Russian Federation

[3] D.I. Mendeleev University of Chemical Technology of Russia, Higher Chemical College of the Russian Academy of Sciences, 9 Miusskaya Square, Moscow 125047, Russian Federation

[4] N.S. Kurnakov Institute of General and Inorganic Chemistry, Russian Academy of Sciences, GSP-1, Leninsky prospect, 31, 119991 Moscow, Russian Federation

[5] A.V. Nikolaev Institute of Inorganic Chemistry, Siberian Branch of Russian Academy of Sciences, 3 Academician Lavrentiev Ave., Novosibirsk 630090, Russian Federation

[*]Correspondence: mbabak@cityu.edu.hk (M.V.B), fershtat@bk.ru (L.L.F.)

[≠]These authors contributed equally to this work

Keywords: nitrogen heterocycles; furoxan; oxadiazole; malignant pleural mesothelioma; NO donors; antiproliferative activity

Table of Contents

S1. Chemical Experimental Section 2

S1.1. General information 2

S1.2. Synthesis of bis(1,2,4-oxadiazolyl)furoxans 2a-l 2

S1.2.1 Synthesis of 3,4-bis(1,2,4-oxadiazol-3-yl)furoxan (2a): 2

S1.2.2. Synthesis of 3,4-Bis(5-(R)-1,2,4-oxadiazolyl)furoxans (2b-l): 3

S1.3. NO release assay 6

S1.4. NMR spectra 7

S2. Crystallographic data 32

S3. Biological activity 34

S3.1. Cell lines and culture conditions 34

S3.2. Evaluation of anticancer activity 34

S3.3. Evaluation of NO release *in vitro* 34

S3.4. JC1- assay 35

S3.5. ROS detection 35

S3.6. Annexin V/PI assay 36

S4. References 37

S1. Chemical Experimental Section

S1.1. General information

All reactions were carried out in well-cleaned oven-dried glassware with magnetic stirring. ^{1}H, ^{13}C NMR spectra were recorded on a Bruker AM-300 (300.13 and 75.47 MHz, respectively) spectrometer and referenced to residual solvent peak. The chemical shifts are reported in ppm (δ). Mass spectra were measured using a Finnigan MAT INCOS-50 instrument. The IR spectra were recorded on the Simex FT-801 IR-Fourier spectrometer in the 4000-550 cm^{-1} region (spectral resolution 4 cm^{-1}) using the universal optical attenuated total reflection (ATR) accessory with ZnSe crystal plate. ZaIR 3.5 software (Simex) was used to carry out baseline correction and normalization of FEAR spectra. A background (air) measurement was taken for every sample processed. The peaks corresponding to CO_2 vibrations were removed using the "straight line generation" option in the ZaIR 3.5 software (Simex). Raw spectra were preprocessed using a simple two-point linear subtraction baseline correction method. Spectra were the vector normalized. Spectrum smoothing was not performed. High resolution mass spectra were recorded on a Bruker microTOF spectrometer with electrospray ionization (ESI). All measurements were performed in a positive (+MS) ion mode (interface capillary voltage: 4500V) with scan range m/z: 50–3000. External calibration of the mass spectrometer was performed with Electrospray Calibrant Solution (Fluka). A direct syringe injection was used for all analyzed solutions in MeCN or CH_3OH (flow rate: 3 μL min^{-1}). Nitrogen was used as nebulizer gas (0.4 bar) and dry gas (4.0 L·min^{-1}); interface temperature was set at 180 °C. All spectra were processed by using Bruker Data Analysis 4.0 software package. Elemental analyses were performed by the CHN Analyzer Perkin-Elmer 2400. Analytical thin-layer chromatography (TLC) was carried out on Merck 25 TLC silica gel 60 F254 aluminum sheets. The visualization of the TLC plates was accomplished with a UV light. All solvents were purified and dried using standard methods prior to use. All standard reagents were purchased from Aldrich or Acros Organics and used without further purification. Some products were purified via column chromatography. Chromatography was performed on silica gel 60 Å (0.060–0.200 mm, Acros Organics).

S1.2. Synthesis of bis(1,2,4-oxadiazolyl)furoxans 2a-l

S1.2.1 Synthesis of 3,4-bis(1,2,4-oxadiazol-3-yl)furoxan(2a):

Bisamidoxime **1c** (1.5 mmol, 303 mg) and $BF_3{\cdot}OEt_2$ (0.016 mmol, 20 μL) were added to a mixture of trimethyl orthoformate (20 mmol, 2.2 mL) and MeCN (7 mL). The reaction mixture was stirred for 8 h at room temperature until the formation of precipitate and consumption of substrate **1c** (TLC monitoring, eluent $CHCl_3$/ EtOAc, 1:1). Then the reaction mixture was poured

onto ice, the precipitate formed was filtered off, washed with cold water (2x20 mL), and dried in air.

3,4-Bis(1,2,4-oxadiazolyl)furoxan, 2a:

yield 223 mg (67%), white powder; R_f (15 $CHCl_3$: 1 EtOAc) = 0.5. ^{1}H NMR (300 MHz, DMSO-[d_6]) *δ, ppm*: 10.06 (s, 1H), 9.98 (s, 1H); ^{13}C NMR (75 MHz, DMSO-[d_6]) *δ, ppm*: 169.2, 168.9, 158.1, 156.1, 145.9, 106.8. IR (KBr), *ν*: 3109, 1667, 1547, 1530, 1364, 1271, 1092, 1019, 984, 942, 995, 894, 819, 734, 611 cm^{-1}. HRMS (ESI) calcd. for $C_6H_2N_6O_4Na^+$: 245.0030. Found: 245.0036 $[M+Na]^+$.

S1.2.2. Synthesis of 3,4-Bis(5-(R)-1,2,4-oxadiazolyl)furoxans (2b-l):

1,1'-Carbonyldiimidazole (CDI) (2.1 mmol, 340 mg) was added to a solution of the corresponding carboxylic acid (1.05 mmol) in acetonitrile (10 mL). The reaction mixture was stirred at room temperature for 1 h. Then bis(amidoxime) **1** (1 mmol, 202 mg) was added to thus formed acylimidazole. The resulting reaction mixture was additionally stirred for 3 h until the complete formation of acylated intermediates (TLC monitoring). Then 1,4-diazabicyclo[2.2.2]octane (DABCO) (4 mmol, 448 mg) was added. The reaction mixture was heated to 50°C and stirred for 12-52 h until complete formation of cyclization products (**2a-k**). Then the reaction mixture was poured onto ice, the precipitate formed was filtered off and dried in air.

3,4-Bis(5-(4-nitrophenyl)-1,2,4-oxadiazolyl)furoxan, 2b:

yield 413 mg (89%), cream solid; R_f (5 $CHCl_3$: 1 EtOAc) = 0.65. ^{1}H NMR (300 MHz, DMSO-[d_6]) *δ*, ppm: 8.52–8.37 (m, 4H); ^{13}C NMR (75 MHz, DMSO-[d_6]) *δ, ppm*: 175.3, 169.2, 160.2, 150.8, 143.8, 137.6, 132.4, 130.3, 130.2, 128.2 ,128.1, 125.2, 124.4, 107.1. IR (KBr), *ν*: 1615, 1570, 1525, 1464, 1389, 1347, 1269, 1103, 1032, 976, 854, 719 cm^{-1}. HRMS (ESI) calcd. for $C_{18}H_8N_8O_8Na^+$: 487.0357. Found: 487.0363 $[M+Na]^+$.

3,4-Bis(5-(3-nitrophenyl)-1,2,4-oxadiazolyl)furoxan, 2c:

yield 399 mg (86%), yellow solid; R_f (5 $CHCl_3$: 1 EtOAc) = 0.65. ^{1}H NMR (300 MHz, DMSO-[d_6]) *δ*, ppm: 8.83 (s, 1H), 8.71 (s, 1H), 8.66 – 8.49 (m, 4H), 7.97 (dt, *J* = 12.1, 8.0 Hz, 2H); ^{13}C NMR (75 MHz, DMSO-[d_6]) *δ, ppm*: 175.4, 175.0, 159.4, 157.6, 148.7, 148.7, 145.8, 134.6, 134.5, 132.2, 132.2, 128.7, 128.5, 124.4, 124.2 ,123.2, 123.1, 106.7. IR (KBr), *ν*: 1618, 1528, 1446, 1350, 1290, 1125, 972, 938, 817, 740, 719 cm^{-1}. HRMS (ESI) calcd. for $C_{18}H_8N_8O_8Na^+$: 487.0357. Found: 487.0349 $[M+Na]^+$.

3,4-Bis(5-(2-nitrophenyl)-1,2,4-oxadiazolyl)furoxan, 2d:

yield 348 mg (75%), yellow solid; R_f (5 $CHCl_3$: 1 EtOAc) = 0.65. ^{1}H NMR (300 MHz, DMSO-[d_6]) *δ*, ppm: 8.30-8.13 (m, 4H), 8.05-7.99 (m, 4H); ^{13}C NMR (75 MHz, DMSO-[d_6]) *δ, ppm*:

174.3, 173.8, 159.3, 157.4, 148.5, 145.6, 144.2, 135.3, 135.3, 134.5, 134.4, 132.4, 132.3, 125.6, 125.5 ,117.0, 116.7, 106.8. IR (KBr), *v*: 1615, 1570, 1525, 1389, 1347, 1269, 1103, 869, 854, 734, 719 cm^{-1}. HRMS (ESI) calcd. for $C_{18}H_9N_8O_8^+$: 465.0538, for $C_{18}H_{12}N_9O_8^+$: 482.0793, for $C_{18}H_8N_8O_8Na^+$: 487.0357. Found: 465.0529 [M+H]$^+$, 482.0792 [M+NH$_4$]$^+$, 487.0351 [M+Na]$^+$.

3,4-Bis(5-phenyl-1,2,4-oxadiazolyl)furoxan, 2e:

yield 275 mg (74%), grey powder; R$_f$ (1 $CHCl_3$: 1 EtOAc) = 0.74. ^{1}H NMR (300 MHz, DMSO-[d$_6$]) *δ, ppm*: 8.20 (d, *J* = 7.6 Hz, 2H), 8.12 (d, *J* = 7.6 Hz, 2H), 7.82–7.63 (m, 6H); ^{13}C NMR (75 MHz, DMSO-[d$_6$]) *δ, ppm*: 177.0, 176.8, 159.5, 157.5, 146.1, 134.9, 134.6, 130.4, 130.3, 128.7, 128.6, 122.9, 122.8, 106.8. IR (KBr), *v*: 1605, 1555, 1489, 1448, 1390, 1268, 1220, 1110, 1029, 975, 946, 810, 750, 713 cm^{-1}.HRMS (ESI) calcd. for $C_{18}H_{11}N_6O_4^+$: 375.0836; for $C_{18}H_{10}N_6O_4Na^+$: 397.0656. Found: 375.0830 [M+H]$^+$, 397.0650 [M+Na]$^+$.

3,4-Bis(5-(pyridin-4-yl)-1,2,4-oxadiazolyl)furoxan, 2f:

yield 158 mg (42%), white powder; R$_f$ (15 $CHCl_3$: 1 EtOAc)= 0.30. ^{1}H NMR (300 MHz, DMSO-[d$_6$]) *δ, ppm*: 8.95 (dd, *J* = 6.0, 1.5 Hz, 2H), 8.91 (dd, *J* = 6.1, 1.7 Hz, 2H), 8.13 (dd, *J* = 6.1, 1.6 Hz, 2H), 8.05 (dd, *J* = 6.1, 1.7 Hz, 2H); ^{13}C NMR (75 MHz, DMSO-[d$_6$]) *δ, ppm*: 175.6, 175.3, 159.7, 157.7, 151.8, 151.7, 145.8, 130.0, 129.9, 121.9, 121.8, 106.7. IR (KBr), *v*: 1629, 1605, 1551, 1489, 1448, 1390, 1267, 1220, 1111, 1051, 1030, 975, 946, 811, 751, 712, 683 cm^{-1}. HRMS (ESI) calcd. for $C_{16}H_9N_8O_4^+$: 377.0741. Found: 377.0733 [M+H]$^+$.

3,4-Bis(5-(methoxymethyl)-1,2,4-oxadiazolyl)furoxan, 2g:

yield 239 mg (77%), yellow oil; R$_f$ (5 $CHCl_3$: 1 EtOAc) = 0.7. ^{1}H NMR (300 MHz, DMSO-[d$_6$]) *δ, ppm*: 4.85 (d, *J* = 13.2 Hz, 4H), 3.44 (d, *J* = 9.3 Hz, 6H); ^{13}C NMR (75 MHz, DMSO-[d$_6$]) *δ, ppm*: 178.0, 177.8, 158.2, 156.1, 145.3, 105.8, 64.3, 64.3, 58.9, 58.8. IR (KBr), *v*: 2938, 2837, 1621, 1560, 1526, 1389, 1345, 1268, 1110, 972, 946, 918, 813, 714 cm^{-1}. HRMS (ESI) calcd. for $C_{10}H_{11}N_6O_6^+$: 311.0735; for $C_{10}H_{10}N_6O_6Na^+$: 333.0554. Found: 311.0741 [M+H]$^+$; 333.0556 [M+Na]$^+$.

3,4-Bis(5-(*p*-tolyl)-1,2,4-oxadiazolyl)furoxan, 2h:

yield 290 mg (72%), cream solid; R$_f$ (15 $CHCl_3$: 1 EtOAc) = 0.87; ^{1}H NMR (300 MHz, DMSO-[d$_6$]) *δ, ppm*: 8.06 (dd, *J* = 49.2, 7.8 Hz, 4H), 7.50 (dd, *J* = 21.4, 7.9 Hz, 4H), 2.46 (d, *J* = 12,4 Hz, 6H). ^{13}C NMR (75 MHz, DMSO-[d$_6$]) *δ, ppm*: 177.2, 176.9, 159.5, 157.4, 146.8, 146.2, 145.3, 131.2, 130.8, 128.7, 128.6, 120.3, 120.2, 106.7, 21.8, 21.4. IR (KBr), *v*: 1609, 1556, 1498, 1432, 1390, 1276, 1182, 975, 946, 831, 808, 760, 729 cm^{-1}. HRMS (ESI) calcd. for $C_{20}H_{15}N_6O_4^+$: 403.1149, for $C_{20}H_{14}N_6O_4Na^+$: 425.0969, for $C_{20}H_{14}N_6O_4K^+$: 441.0708. Found: 403.1141 [M+H]$^+$, 425.0969 [M+Na]$^+$, 441.0702 [M+K]$^+$.

3,4-Bis(5-(4-methoxyphenyl)-1,2,4-oxadiazolyl)furoxan, 2i:

yield 274 mg (63%), pale yellow solid; R_f (15 $CHCl_3$: 1 EtOAc) = 0.82. ^{1}H NMR (300 MHz, DMSO-[d_6]) *δ, ppm*: 7.65 (d, *J* = 8.2 Hz, 4H), 7.42 (d, *J* = 8.4 Hz, 4H), 2.43 (s, 6H); ^{13}C NMR (75 MHz, DMSO-[d_6]) *δ, ppm*: 176.9, 176.6, 164.2, 164.2, 159.4, 157.3, 146.2, 130.8, 130.7, 115.8, 115.7, 115.2, 115.1, 106.8, 56.3, 56.2. IR (KBr), *ν*: 2966, 2901, 1603, 1497, 1386, 1257, 1171, 1021, 944, 808, 759 cm^{-1}. HRMS (ESI) calcd. for $C_{20}H_{15}N_6O_6^+$: 435.1048 for $C_{20}H_{14}N_6O_6Na^+$: 457.0867, for $C_{20}H_{14}N_6O_6K^+$: 473.0605. Found: 435.1055 [M+H]$^+$, 457.0867 [M+Na]$^+$, 473.0602 [M+K]$^+$.

3,4-Bis(5-(3,4-dimethoxyphenyl)-1,2,4-oxadiazolyl)furoxan, 2j:

yield 351 mg (71%), white solid; R_f (15 $CHCl_3$: 1 EtOAc) = 0.84; ^{1}H NMR (300 MHz, DMSO-[d_6]) *δ, ppm*: 7.82 (d, *J* = 8.3 Hz, 1H), 7.75 (d, *J* = 8.1 Hz, 1H), 7.62 (s, 1H), 7.49 (s, 1H), 7.23 (t, *J* = 9.1 Hz, 2H), 3.91-3.74 (m, 12H); ^{13}C NMR (75 MHz, DMSO-[d_6]) *δ, ppm*: 176.9, 176.7, 159.4, 157.2, 154.1, 154.1, 149.7, 149.6, 146.2, 122.9, 122.8, 114.9, 114.8, 112.7, 110.7, 110.6, 106.8, 56.4, 56.4, 56.2, 55.9. IR (KBr), *ν*: 2836, 1714, 1601, 1555, 1498, 1432, 1390, 1350, 1273, 1225, 1177, 1141, 1098, 1056, 1020, 947, 868, 812, 757 cm^{-1}. HRMS (ESI) calcd. for $C_{22}H_{19}N_6O_8^+$: 495.1259, for $C_{22}H_{18}N_6O_8Na^+$: 517.1078, for $C_{22}H_{18}N_6O_8K^+$: 533.0818. Found: 495.1254 [M+H]$^+$, 517.1070 [M+Na]$^+$, 533.0811 [M+K]$^+$.

3,4-Bis(5-(2-chlorophenyl)-1,2,4-oxadiazolyl)furoxan, 2k:

yield 280 mg (63%), orange powder; R_f (15 $CHCl_3$: 1 EtOAc) = 0.93. ^{1}H NMR (300 MHz, DMSO-[d_6]) *δ, ppm*: 8.15 (dd, *J* = 21.0, 7.9 Hz, 2H), 7.78-7.59 (m, 6H); ^{13}C NMR (75 MHz, DMSO-[d_6]) *δ, ppm*: 175.5, 174.9, 159.2, 157.2, 146.0, 135.5, 135.4, 133.1, 133.0, 132.9, 132.7, 132.1, 132.1, 128.7, 128.6, 122.1, 121.9, 107.0. IR (KBr), *ν*: 2969, 1625, 1595, 1497, 1461, 1427, 1392, 1250, 1103, 1019, 809, 761 cm^{-1}. HRMS (ESI) calcd. for $C_{18}H_9Cl_2N_6O_4^+$: 443.0057 (^{35}Cl), 445.0028 (^{37}Cl); for $C_{18}H_8Cl_2N_6O_4Na^+$: 464.9876 (^{35}Cl), 466.9848 (^{37}Cl). Found: 443.0055 (^{35}Cl), 445.0028 (^{37}Cl) [M+H]$^+$; 464.9869 (^{35}Cl), 466.9837 (^{37}Cl) [M+Na]$^+$.

3,4-Bis-(5-(2-furyl)-1,2,4-oxadiazolyl)furoxan, 2l:

yield 262 mg (74%), yellow solid; R_f (5 $CHCl_3$: 1 EtOAc)= 0.55. ^{1}H NMR (300 MHz, Acetone-[d_6]) *δ, ppm*: 8.07 (ddd, *J* = 18.5, 3.7, 0.8 Hz, 2H), 7.62 (ddd, *J* = 18.5, 3.7, 0.8 Hz, 2H), 6.87 (ddd, *J* = 8.9, 3.6, 1.8 Hz, 2H); ^{13}C NMR (75 MHz, DMSO-[d_6]) *δ, ppm*: 168.6, 168.4, 159.2, 157.2, 150.0, 149.8, 145.8, 138.6, 138.5, 119.7, 119.7, 113.9, 113.9, 106.5. IR (KBr), *ν*: 3124, 1636, 1621, 1539, 1522, 1436, 1404, 1377, 1177, 1106, 1018, 970, 943, 901, 807, 771, 755 cm^{-1}. HRMS (ESI) calcd. for $C_{14}H_7N_6O_6^+$: 355.0422, for $C_{14}H_{10}N_7O_6^+$: 372.0687, for $C_{14}H_6N_6O_6Na^+$:

377.0241, for $C_{14}H_6N_6O_6K^+$: 392.9980. Found: 355.0425 $[M+H]^+$, 372.0687 $[M+NH_4]^+$, 377.0247 $[M+Na]^+$, 392.9983 $[M+K]^+$.

S1.3. NO release assay

The test molecule (0.1 mmol) was dissolved in DMSO (50 mL). 20 μL aliquot of the resulted solution was diluted with phosphate buffer solution (180 μL, pH 7.4, containing 2 μmol *L*-cysteine). The final concentration of the tested compound was $2 \cdot 10^{-4}$ M. The mixture was incubated at 37 °C for 1 h. 50 μL aliquot of the Griess reagent (prepared by mixing sulfanilamide (4 g), *N*-naphthylethylenediamine dihydrochloride (0.2 g) and 85% H_3PO_4 (10 mL) in distilled and deionized water (final volume 100 mL)) was added and incubated for 10 min at 37 °C. UV absorbance at 540 nm was measured using a Multiskan GO Microplate Photometer and calibrated using a standard curve prepared from standard solutions of $NaNO_2$ to give the nitrite concentration. All measurements were made in triplicate.

S1.4. NMR spectra

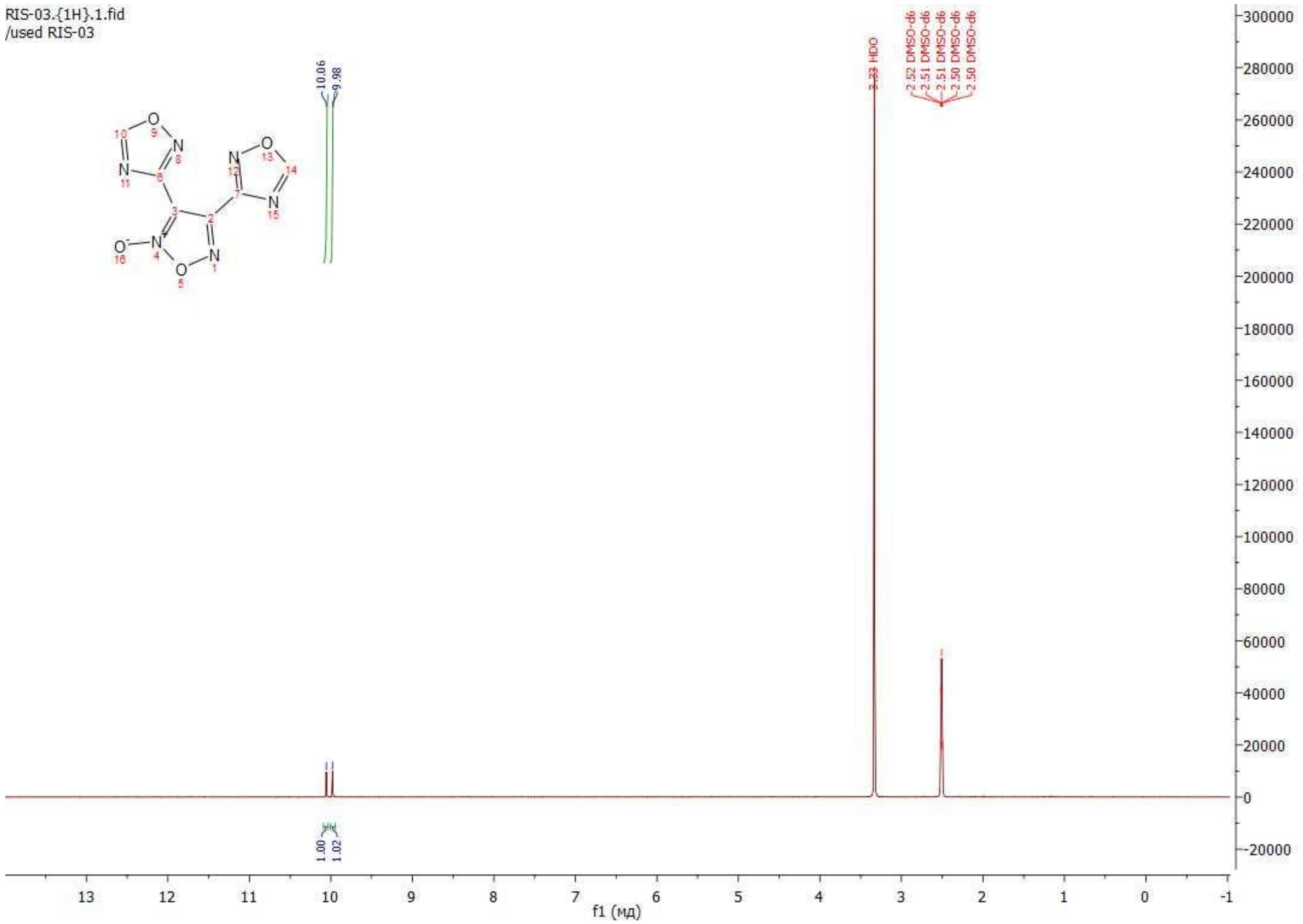

Figure S1. ^{1}H NMR spectrum of **2a**, DMSO-[d_6]

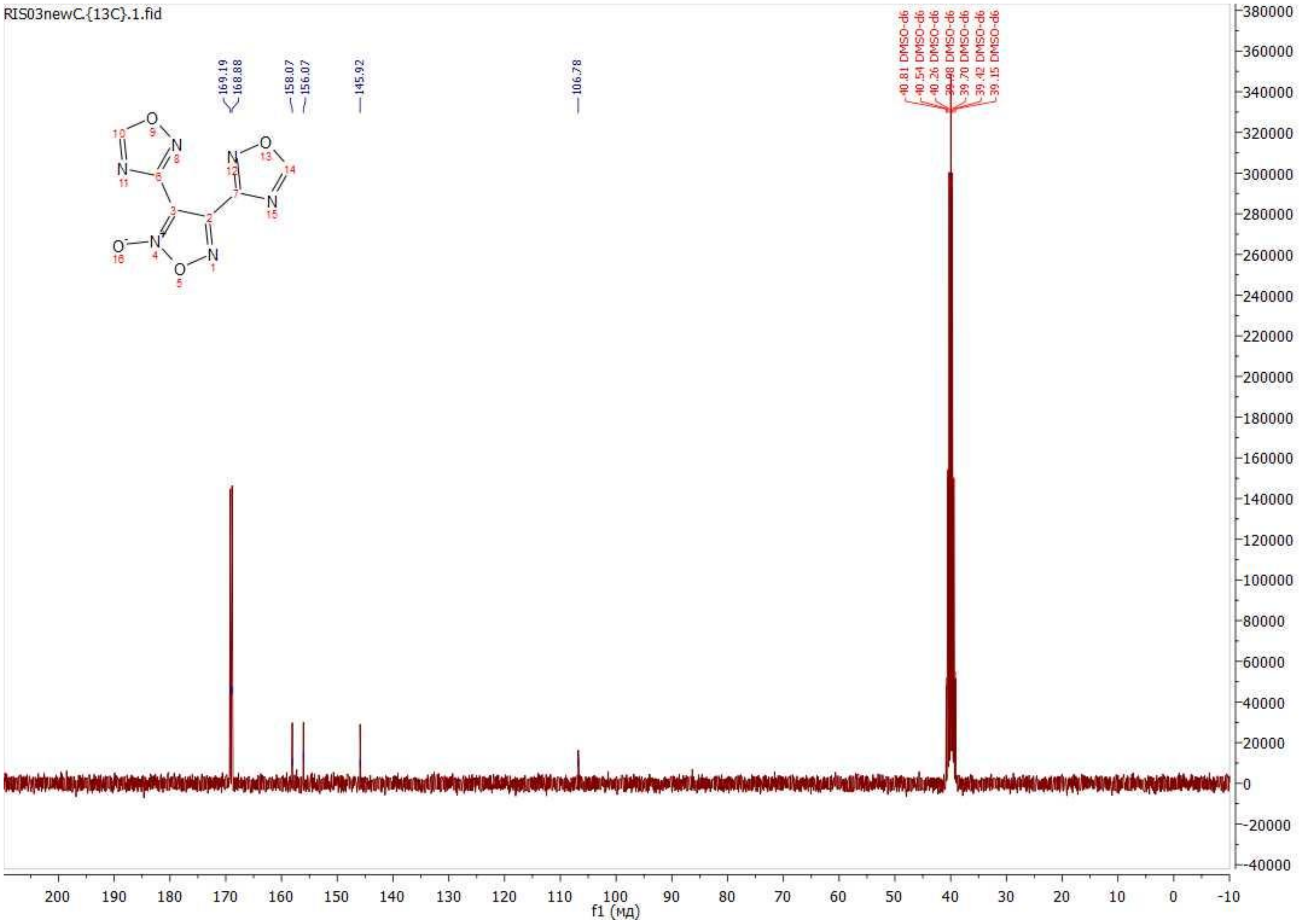

Figure S2. ^{13}C NMR spectrum of **2a**, DMSO-[d_6]

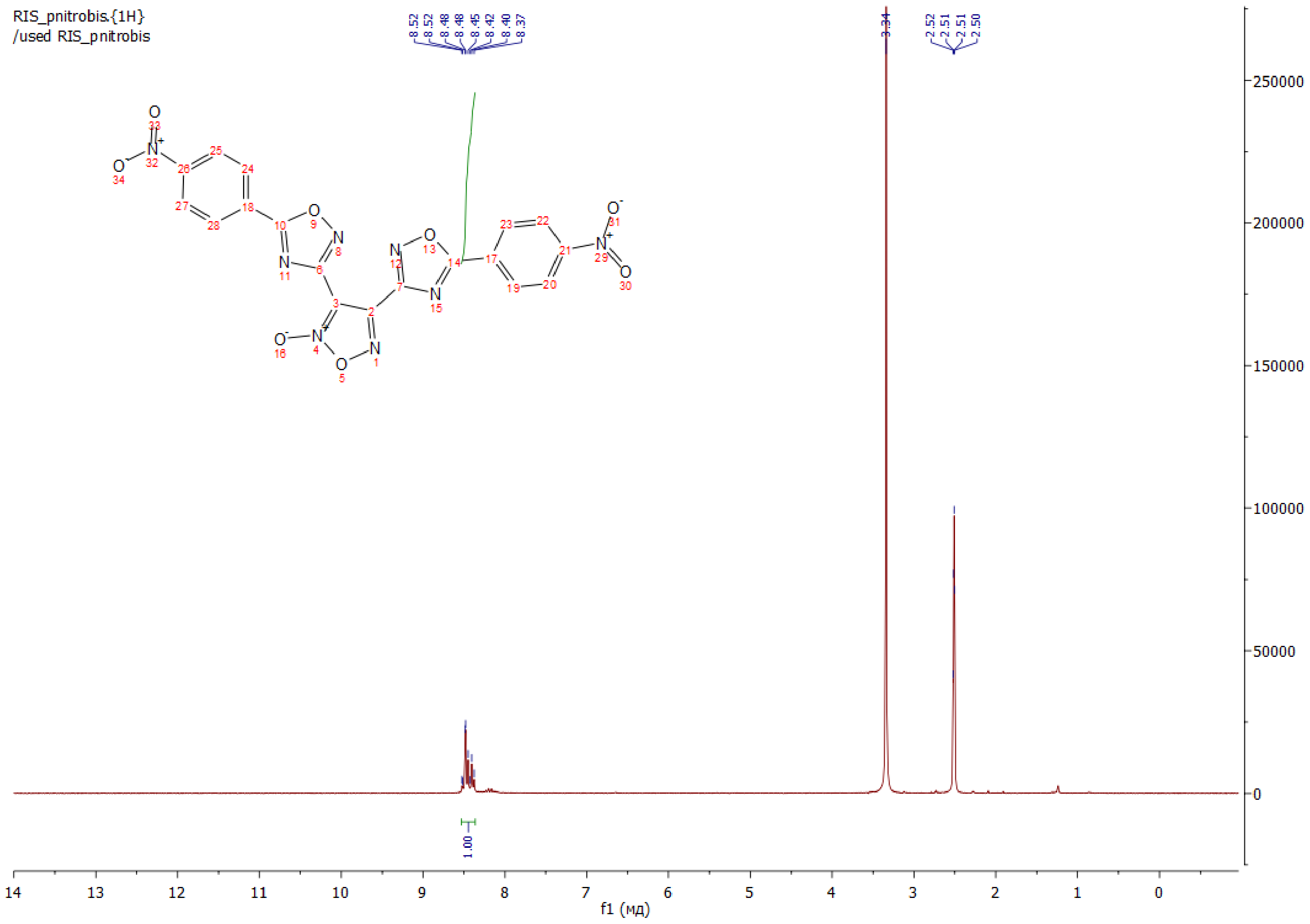

Figure S3. ^{1}H NMR spectrum of **2b**, DMSO-[d$_6$]

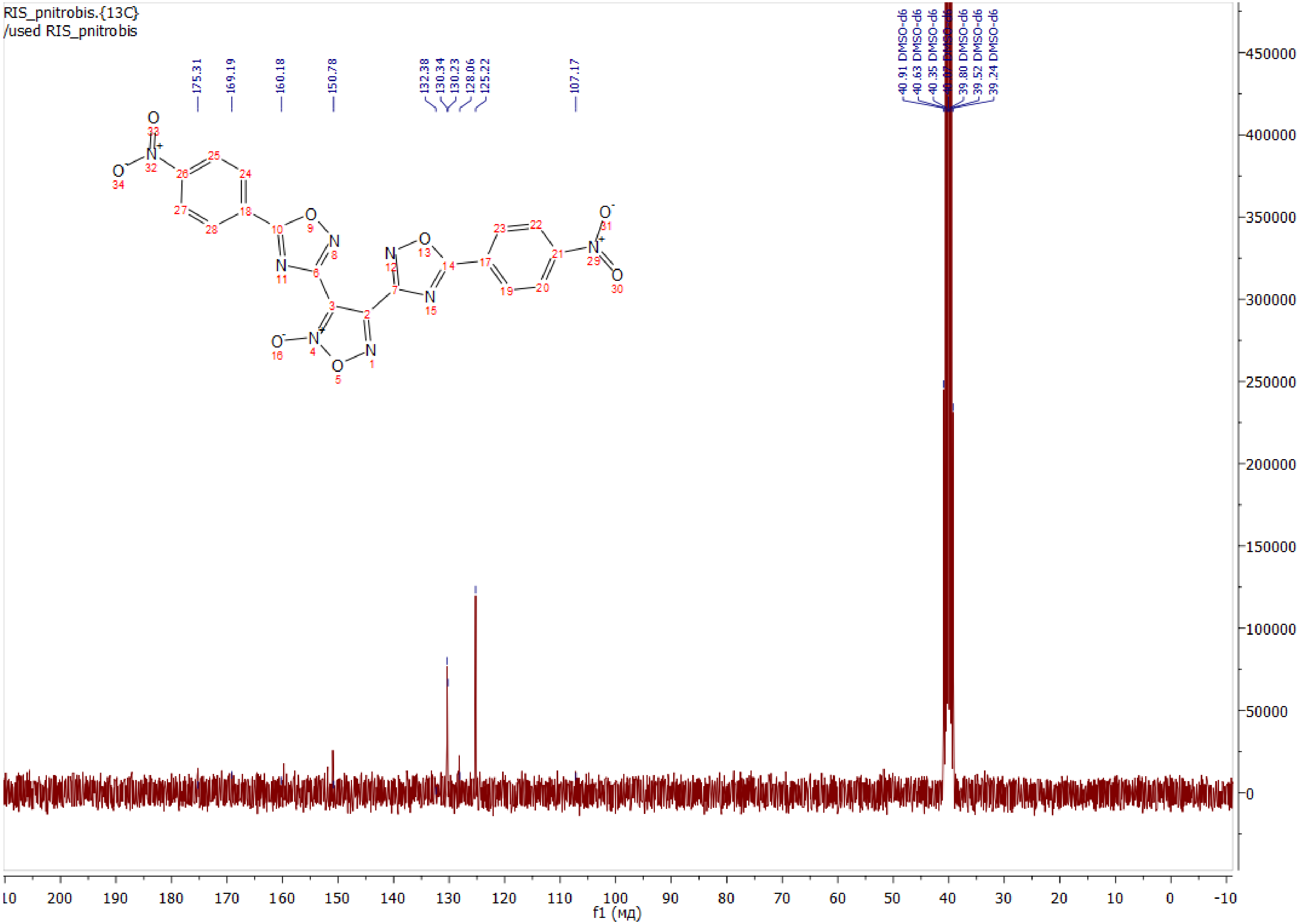

Figure S4. ^{13}C NMR spectrum of **2b**, DMSO-[d_6]

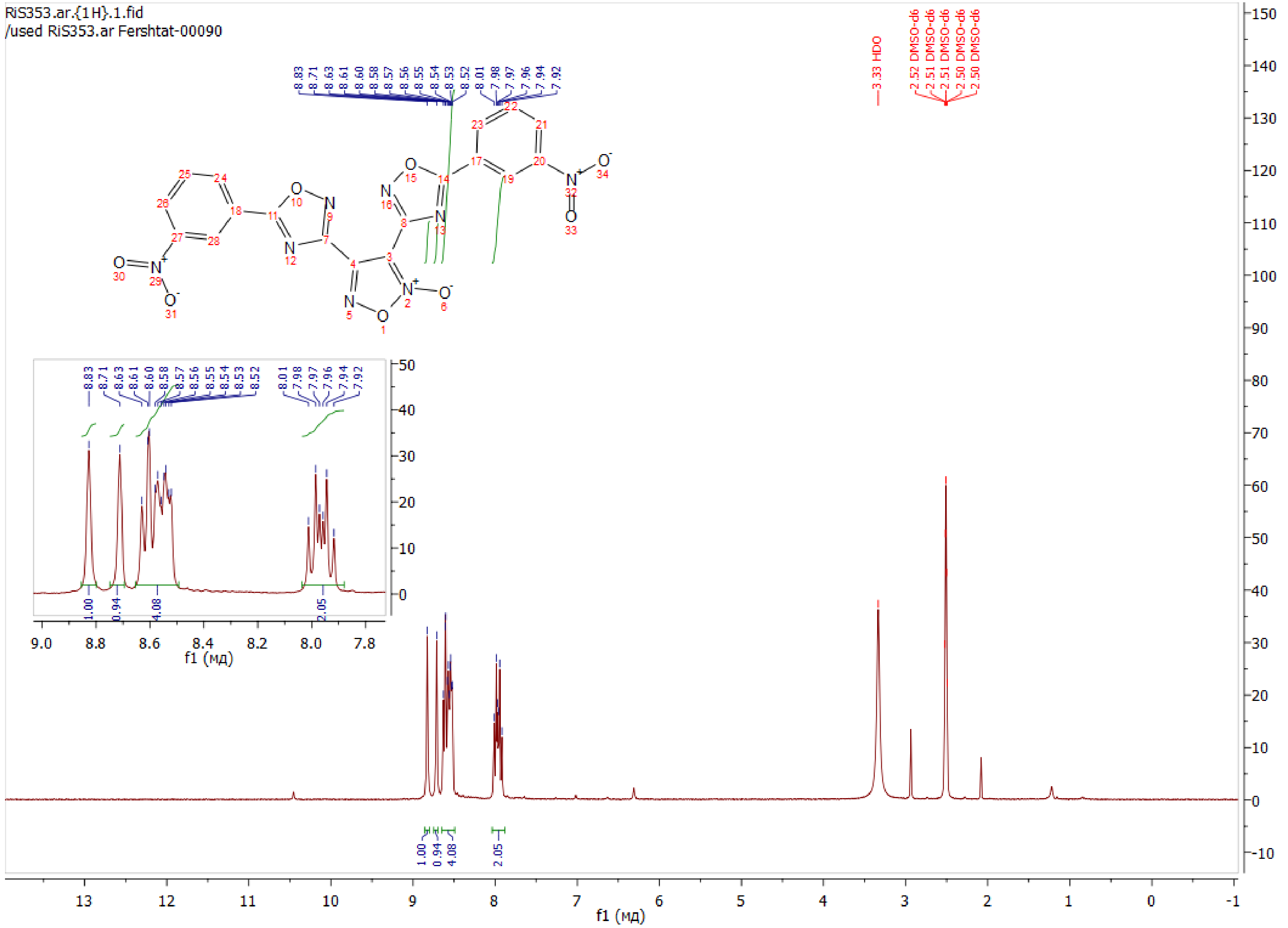

Figure S5. ^{1}H NMR spectrum of **2c**, DMSO-[d_6]

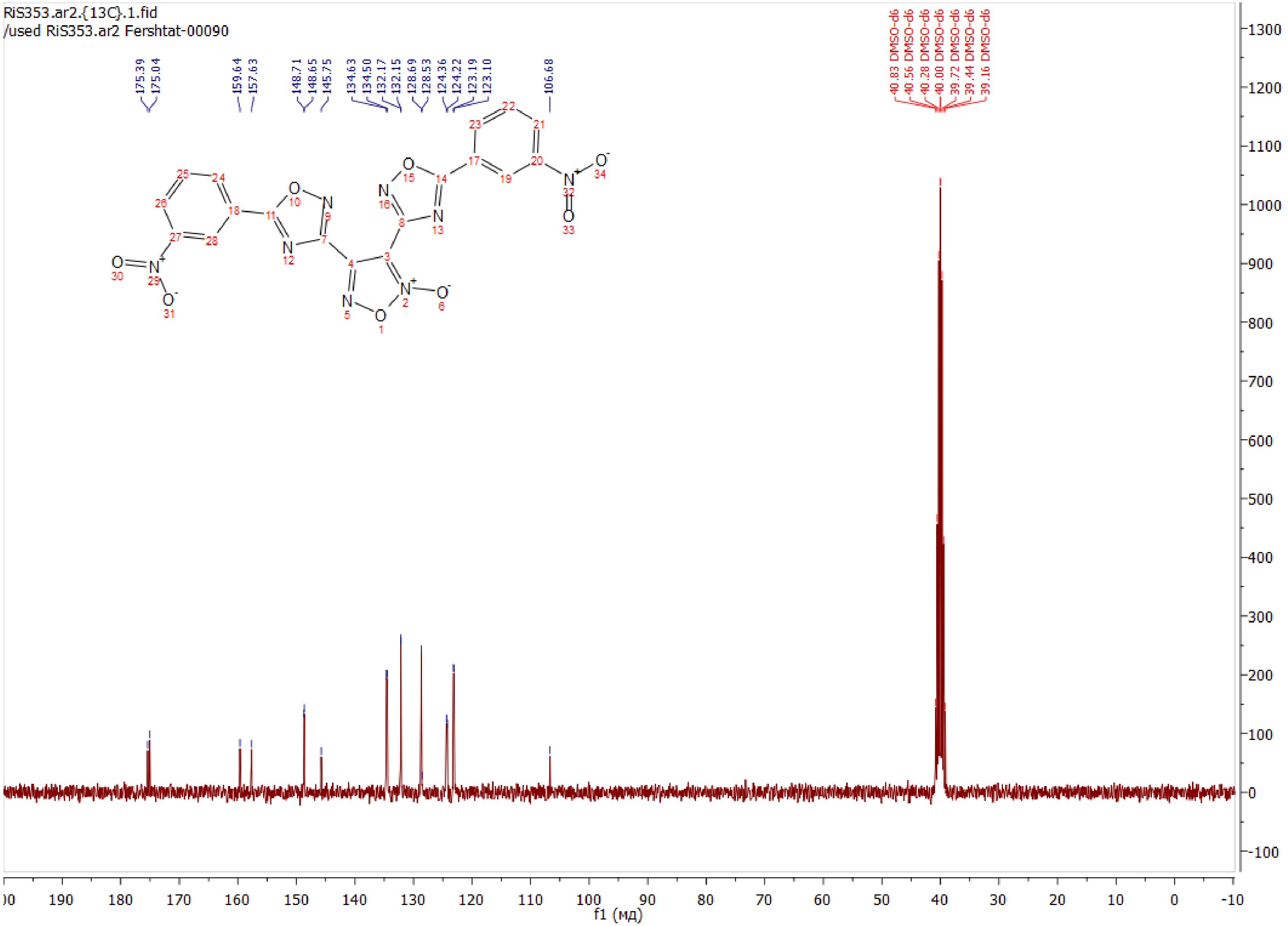

Figure S6. ^{13}C NMR spectrum of **2c**, Acetone-[d$_6$]

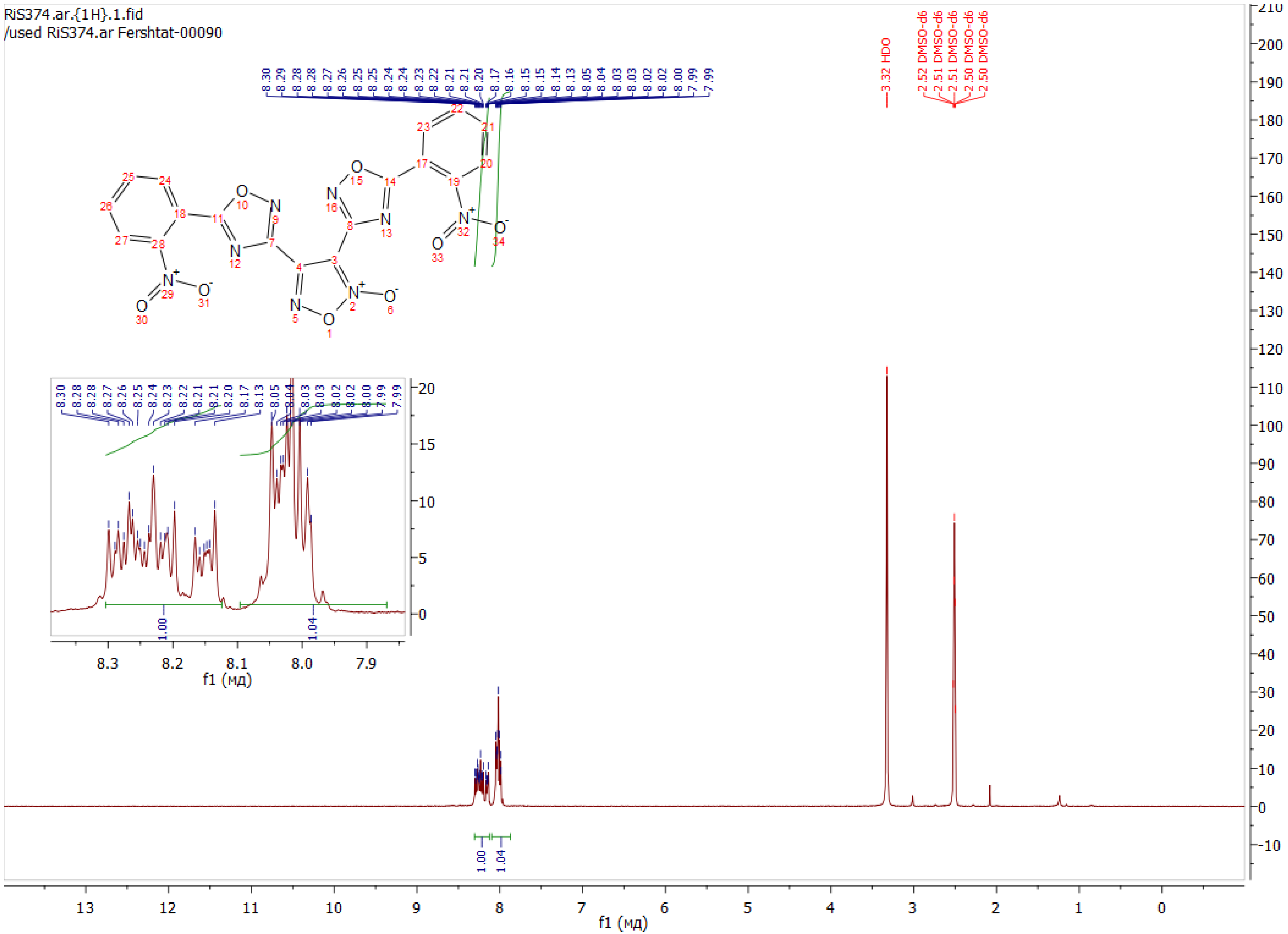

Figure S7. ^{1}H NMR spectrum of **2d**, DMSO-[d_6]

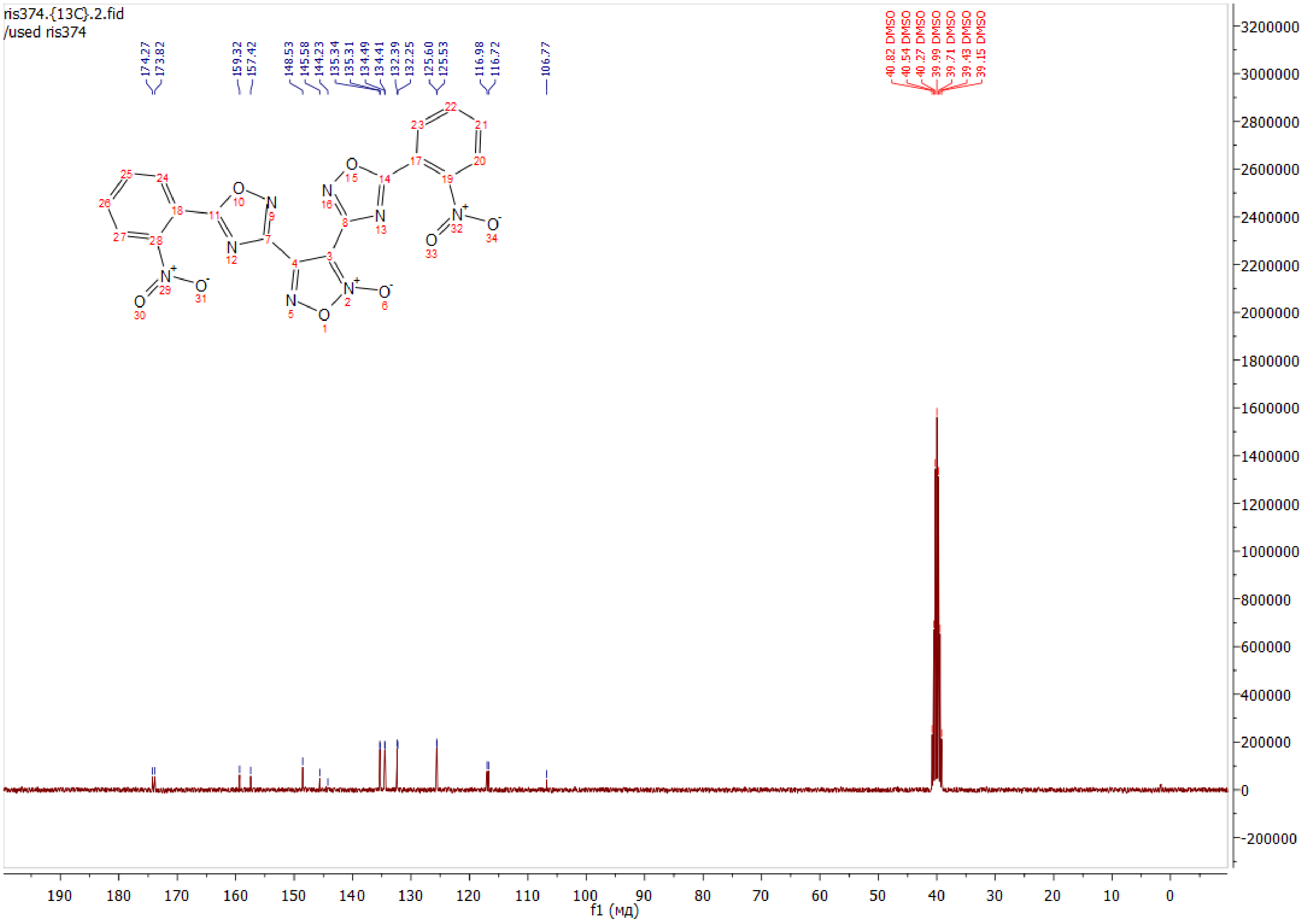

Figure S8. ^{13}C NMR spectrum of **2d**, Acetone-[d$_6$]

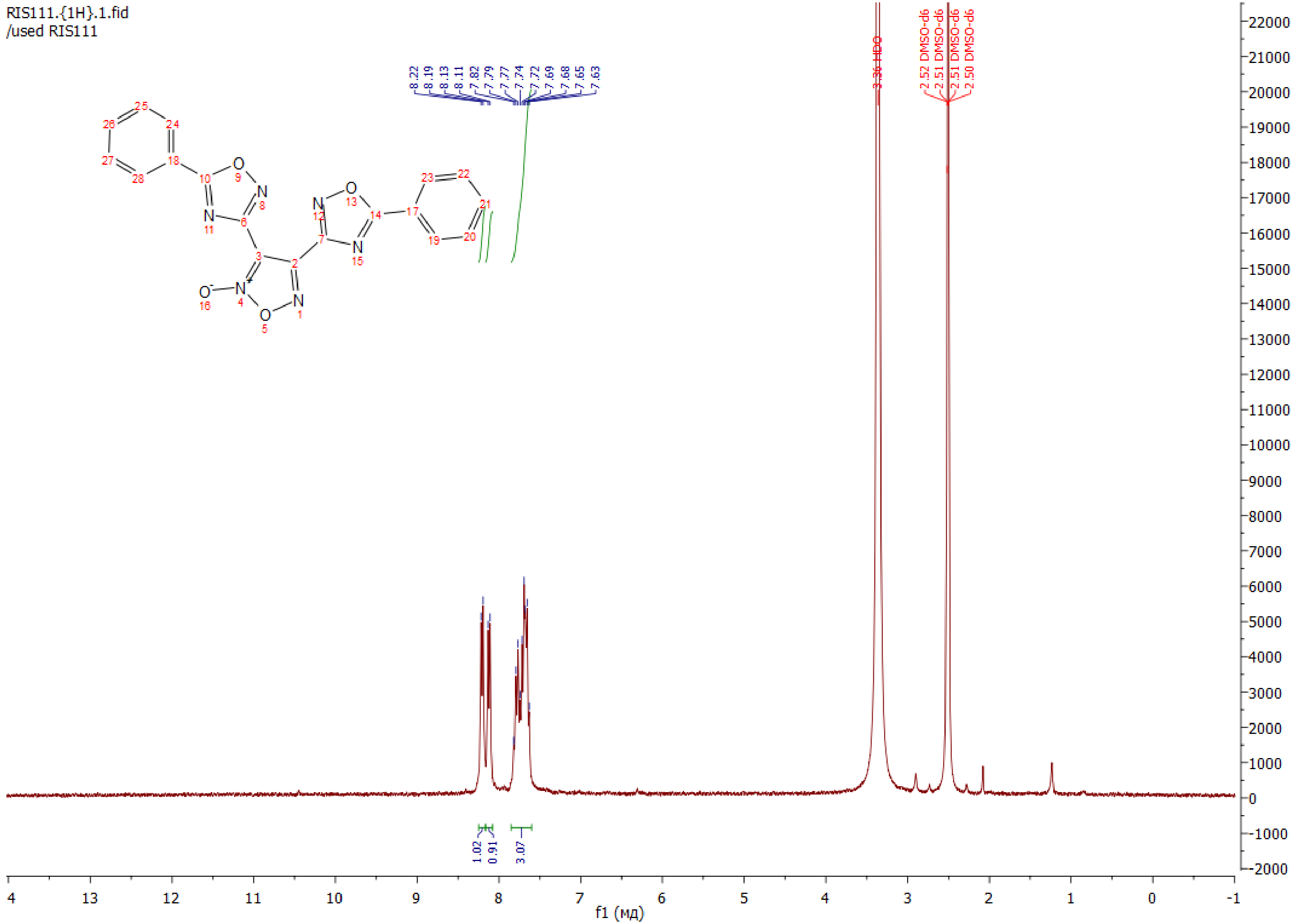

Figure S9. ^{1}H NMR spectrum of **2e**, DMSO-[d_6]

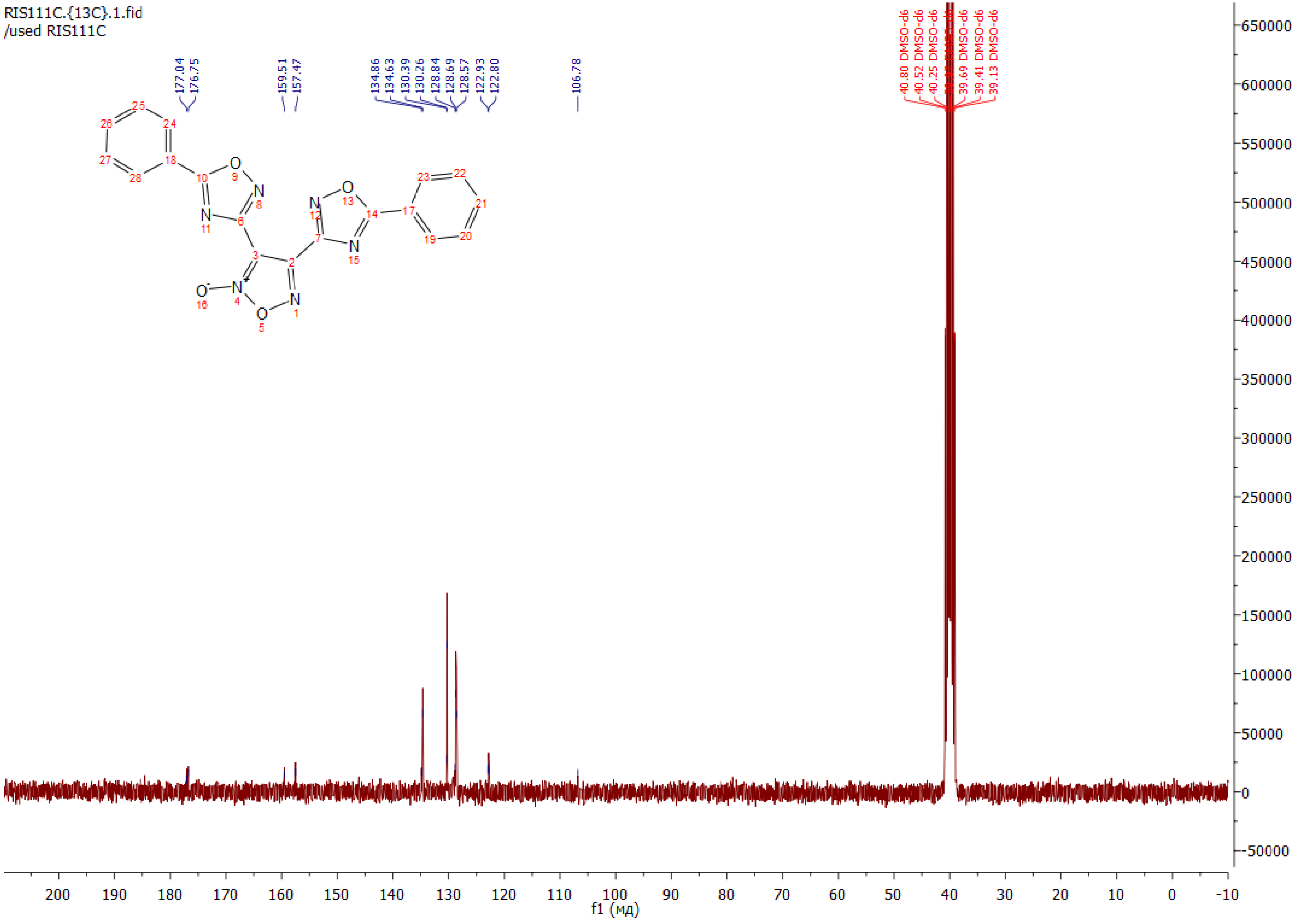

Figure S10. ^{13}C NMR spectrum of **2e**, DMSO-[d_6]

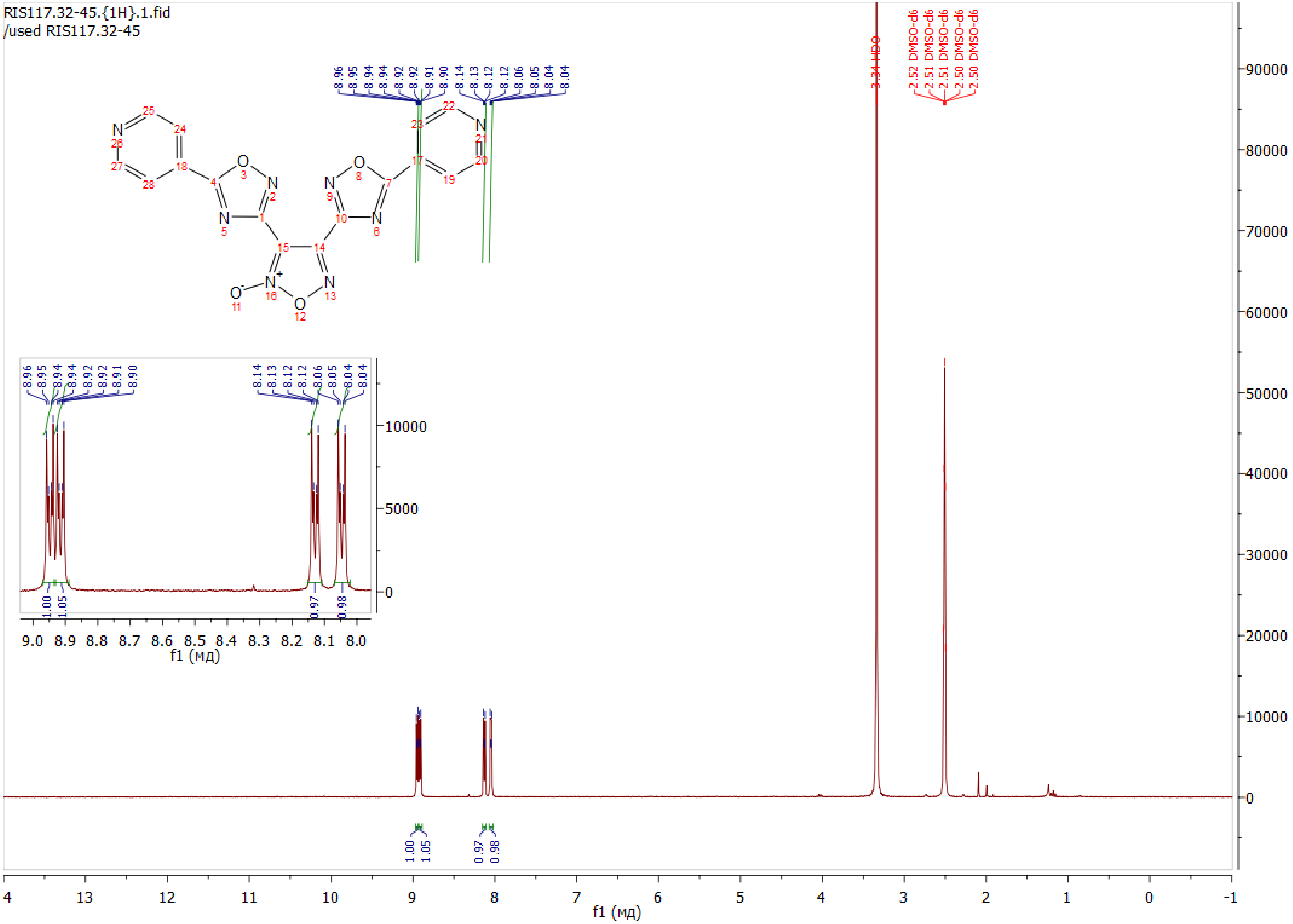

Figure S11. ^{1}H NMR spectrum of **2f**, DMSO-[d$_6$]

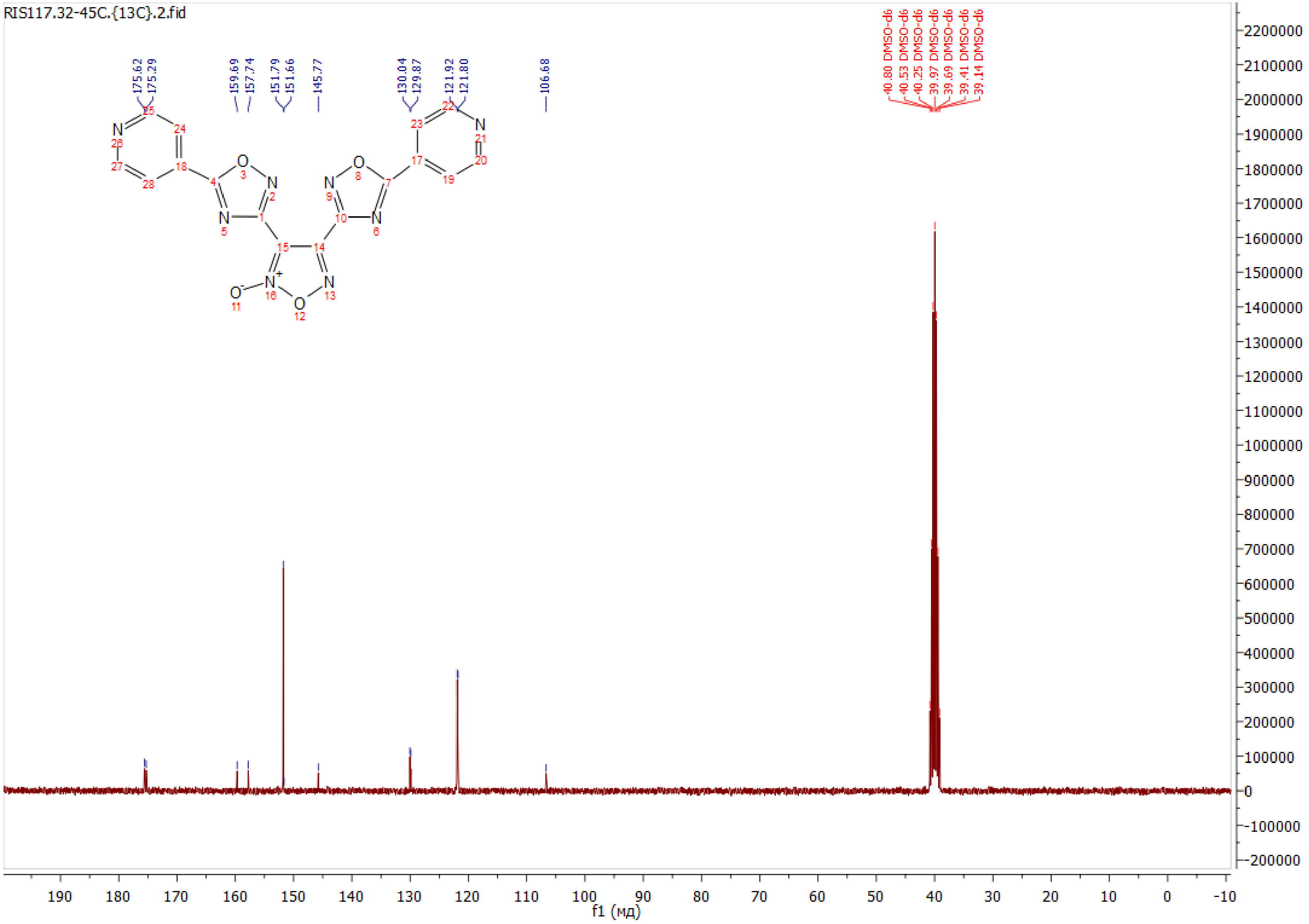

Figure S12. ^{13}C NMR spectrum of **2f**, DMSO-[d_6]

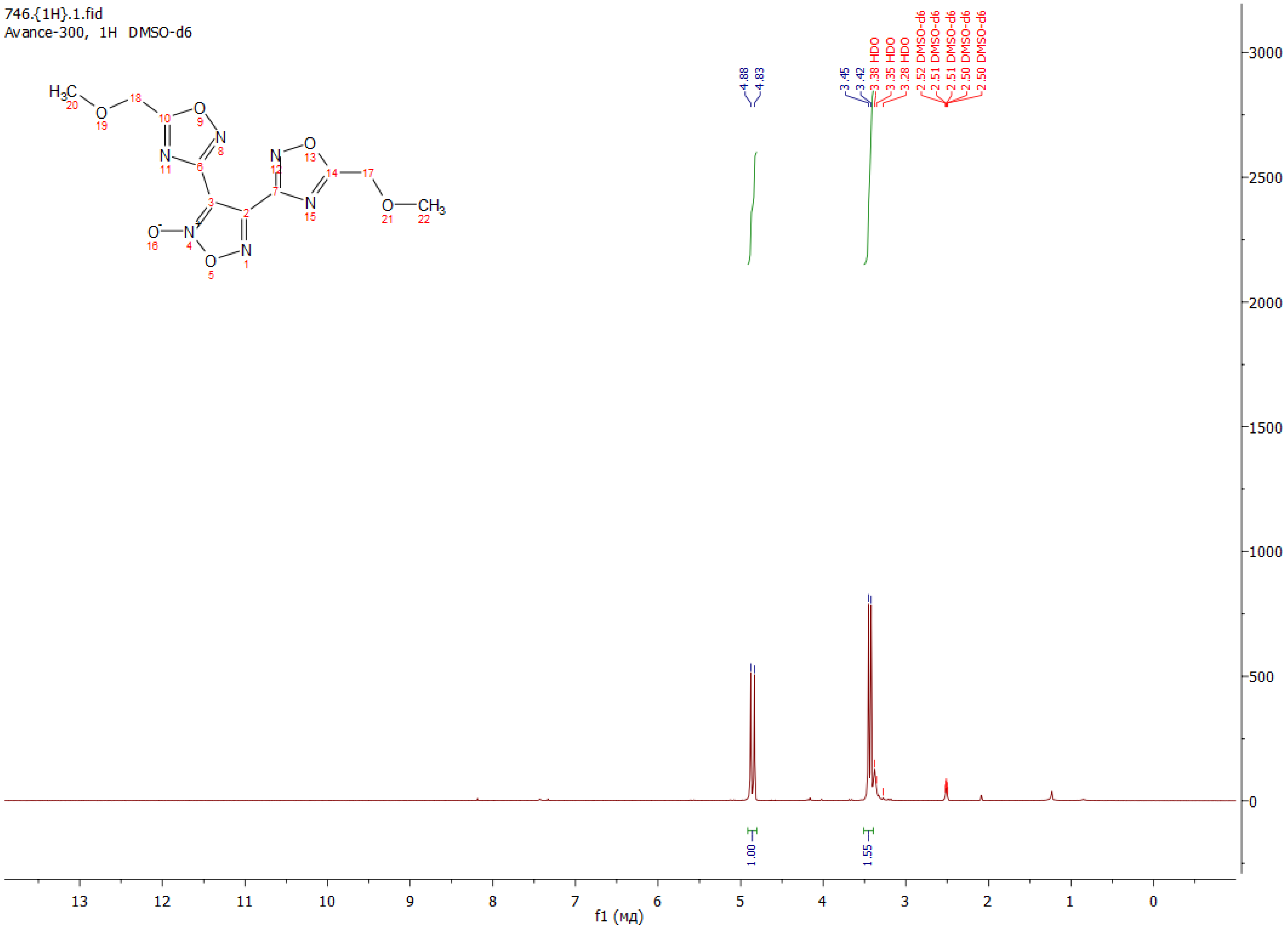

Figure S13. ^{1}H NMR spectrum of **2g**, DMSO-[d_6]

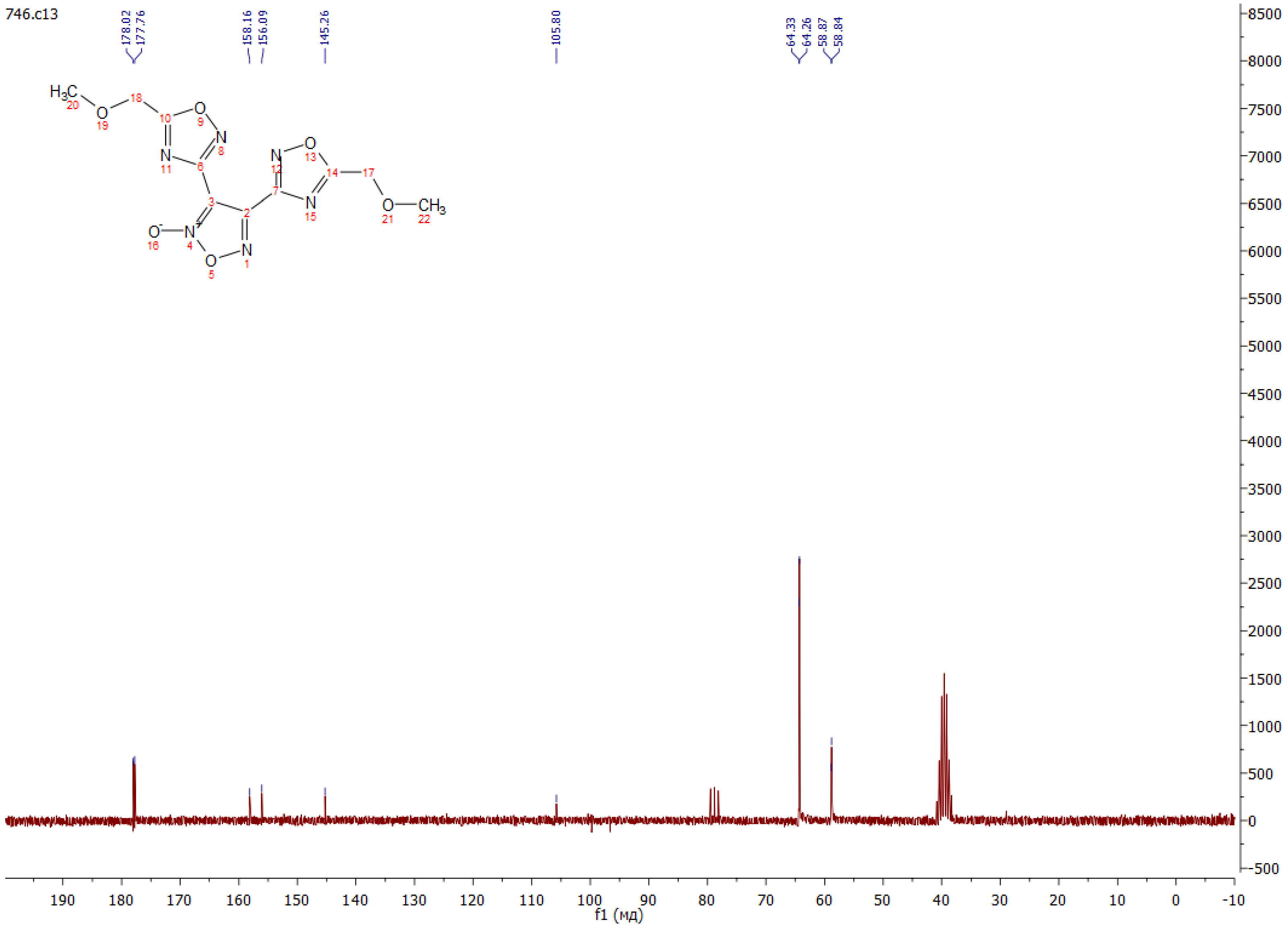

Figure S14. ^{13}C NMR spectrum of **2g**, DMSO-[d_6]

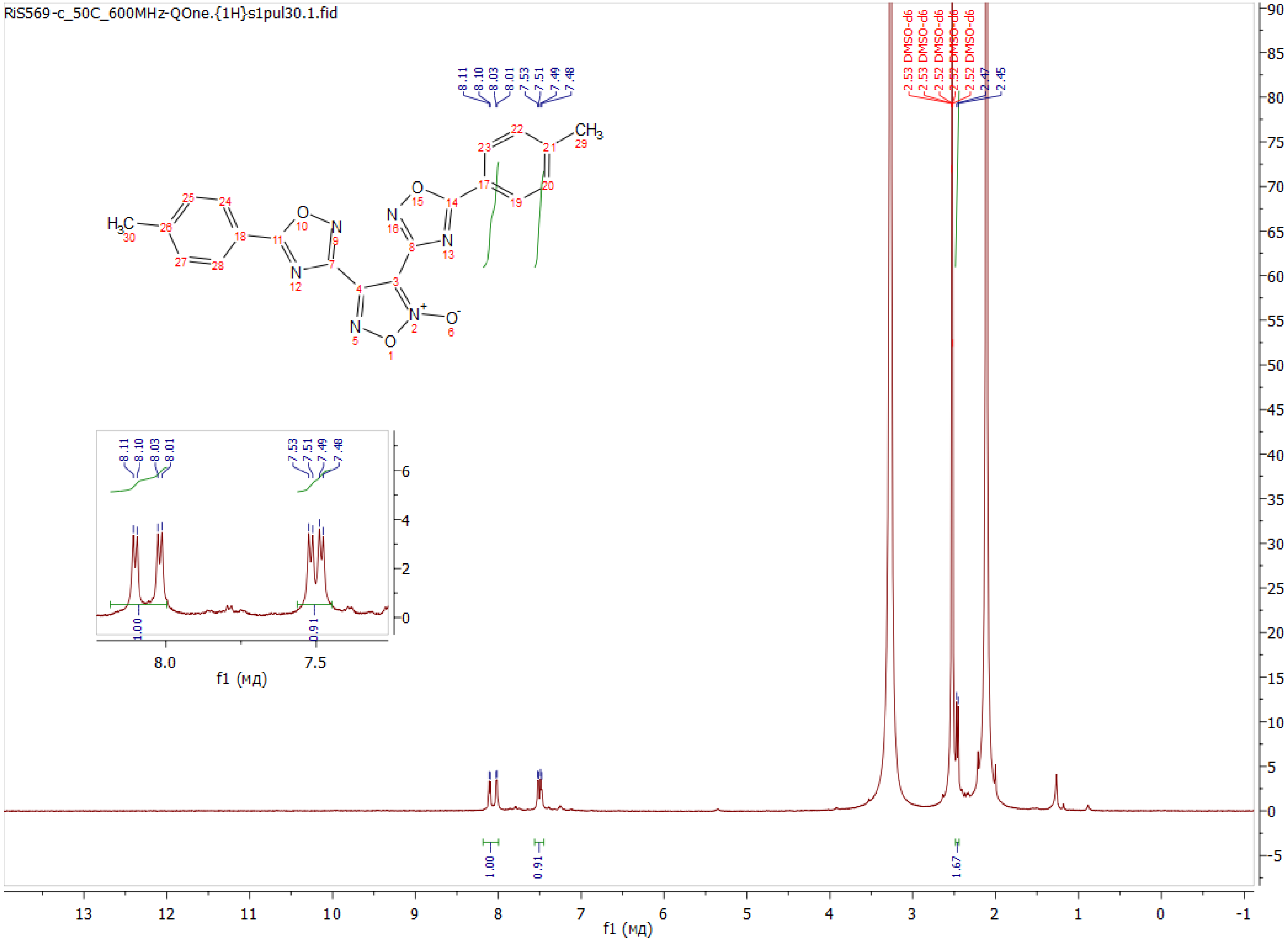

Figure S15. ^{1}H NMR spectrum of **2h**, DMSO-[d$_6$]

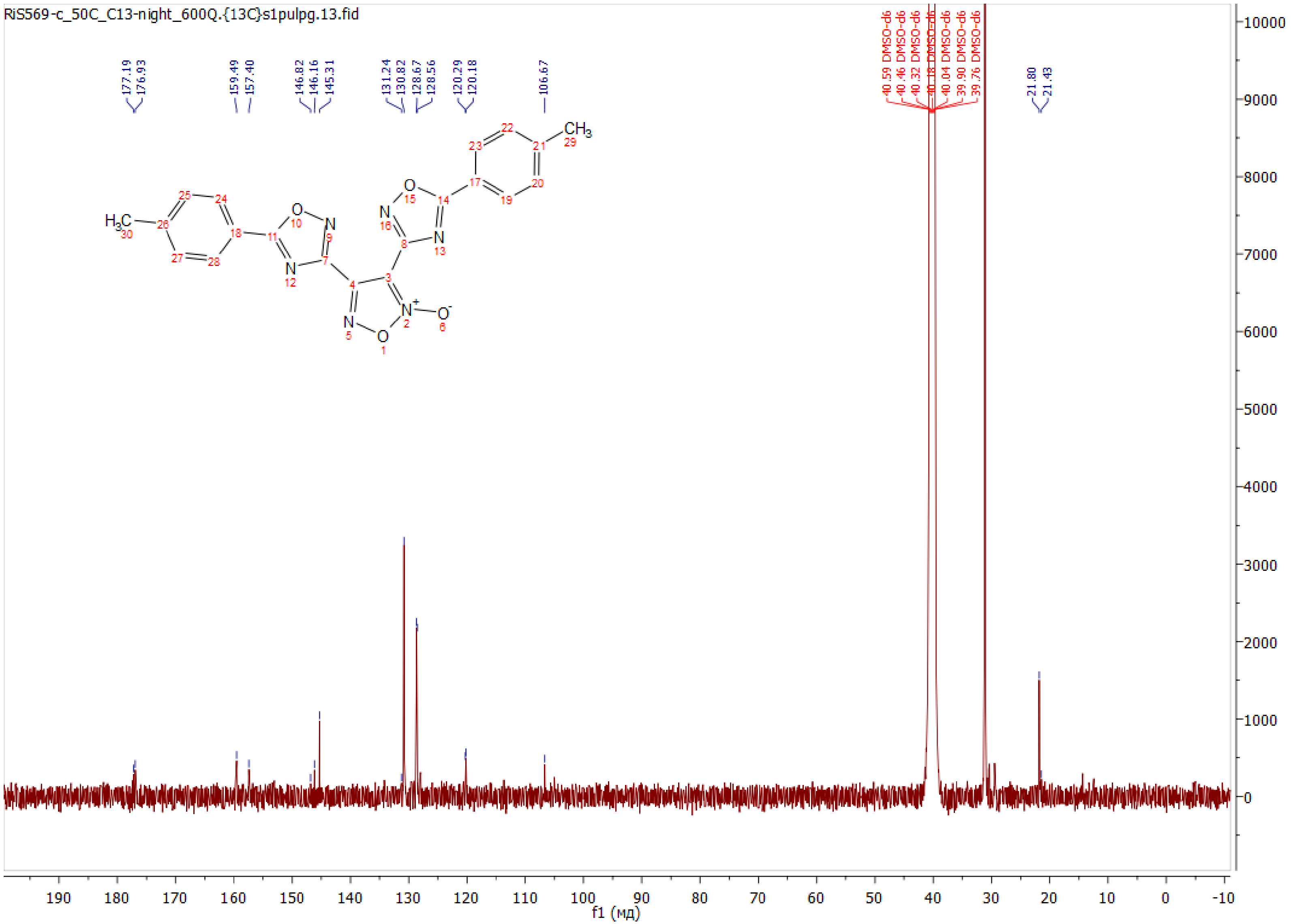

Figure S16. ^{13}C NMR spectrum of **2h**, DMSO-[d_6]

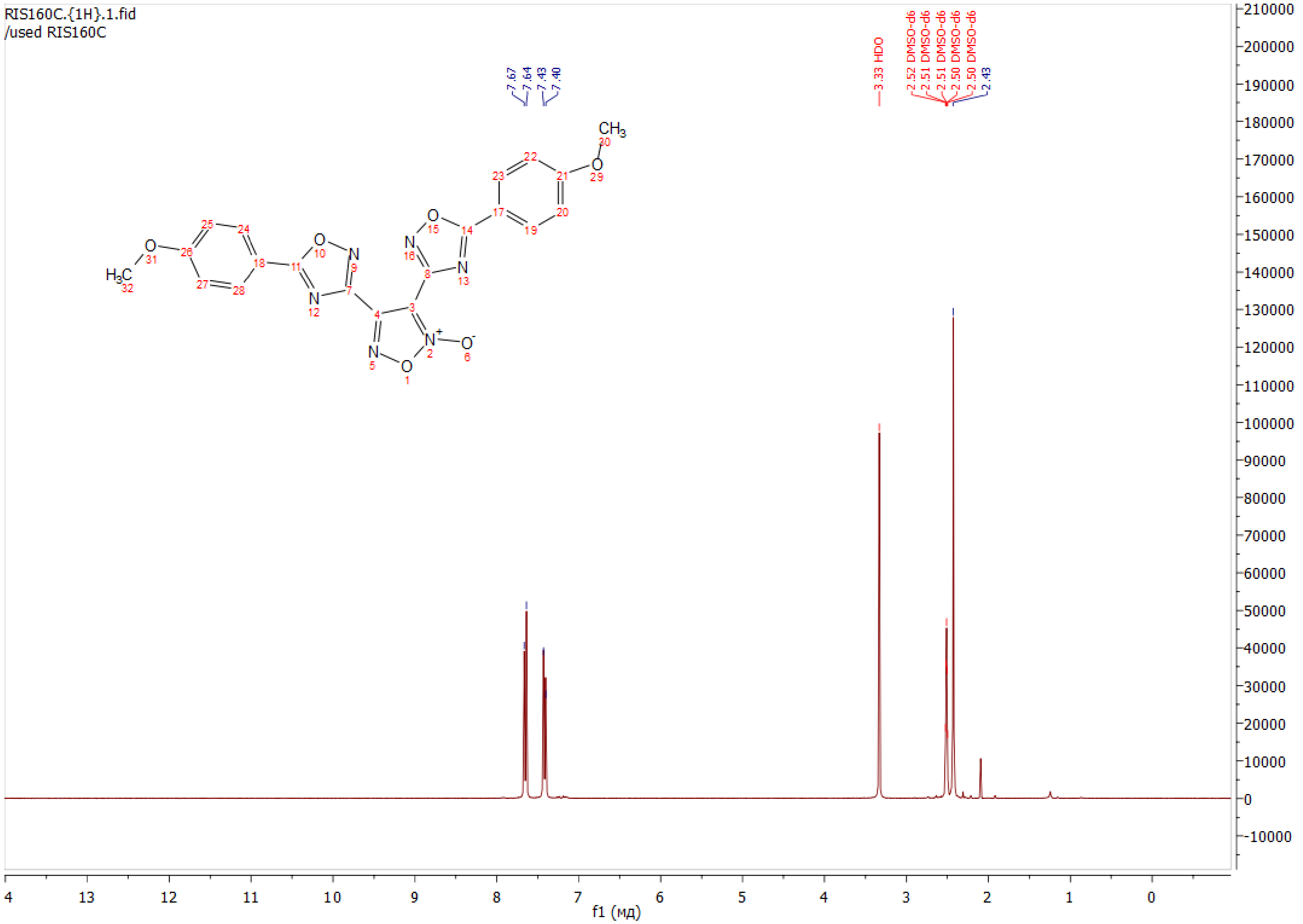

Figure S17. ^{1}H NMR spectrum of **2i**, DMSO-[d$_6$]

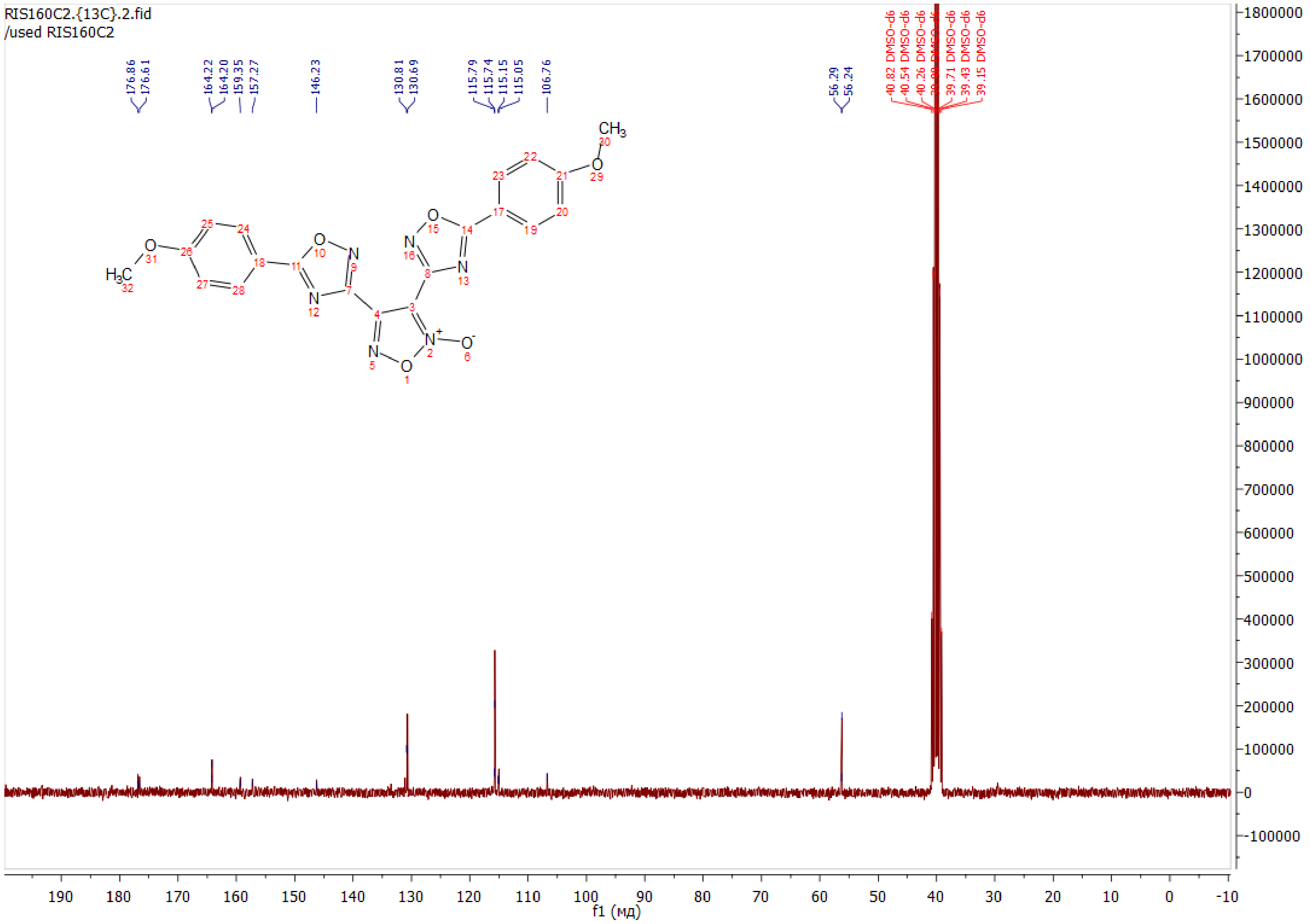

Figure S18. ^{13}C NMR spectrum of **2i**, DMSO-[d_6]

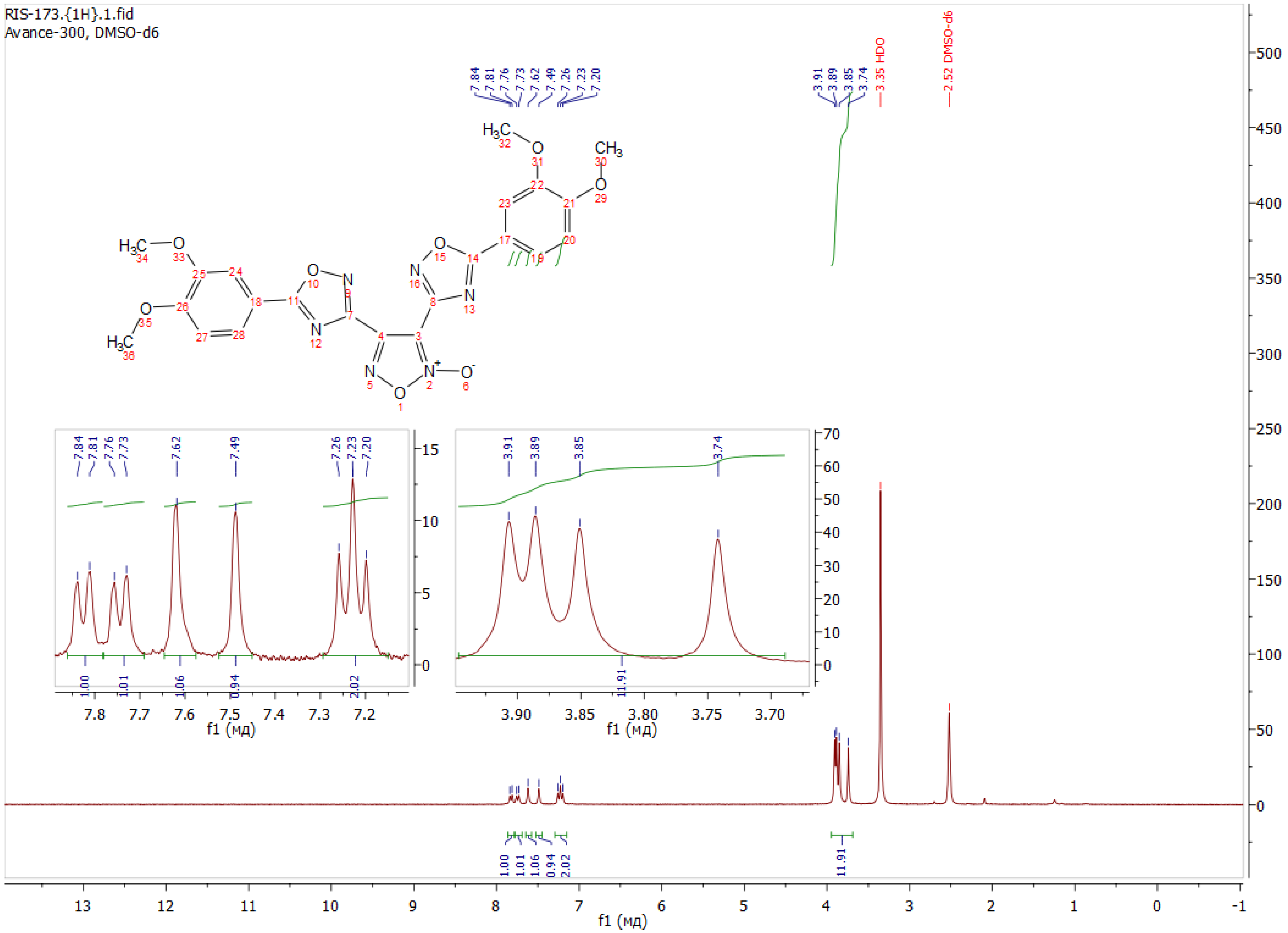

Figure S19. ^{1}H NMR spectrum of **2j**, DMSO-[d_6]

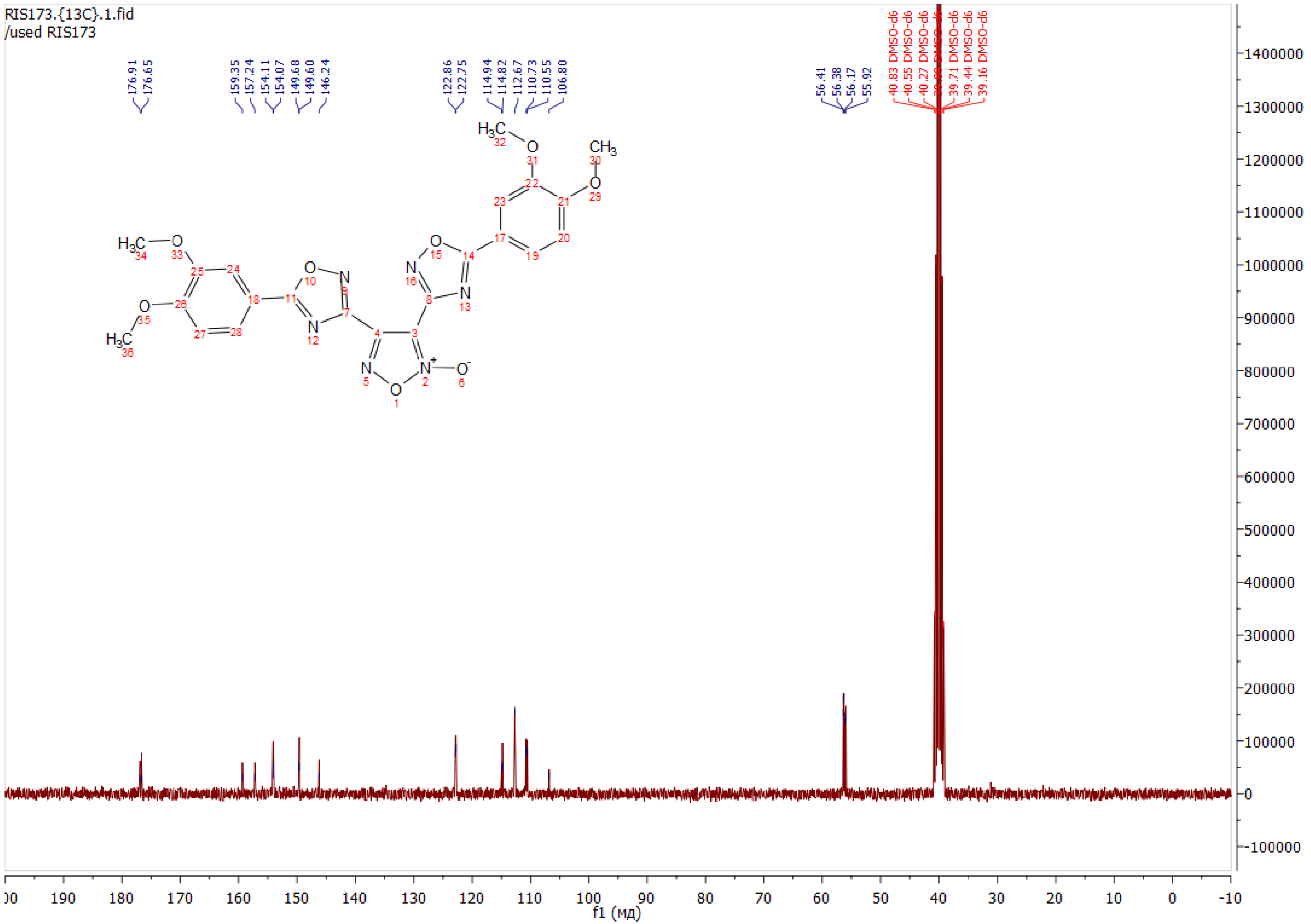

Figure S20. ^{13}C NMR spectrum of **2j**, DMSO-[d_6]

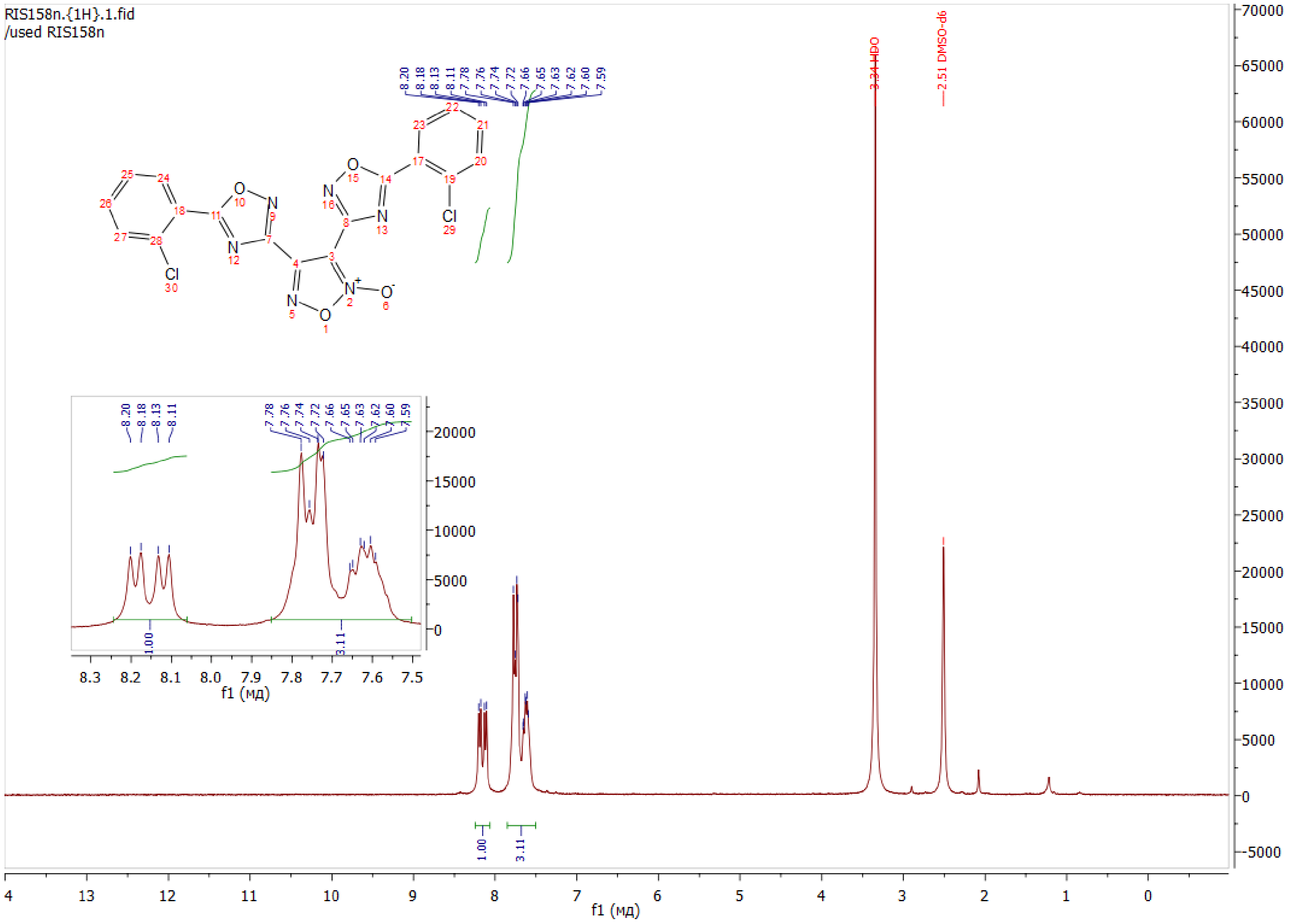

Figure S21. ^{1}H NMR spectrum of **2k**, DMSO-[d$_6$]

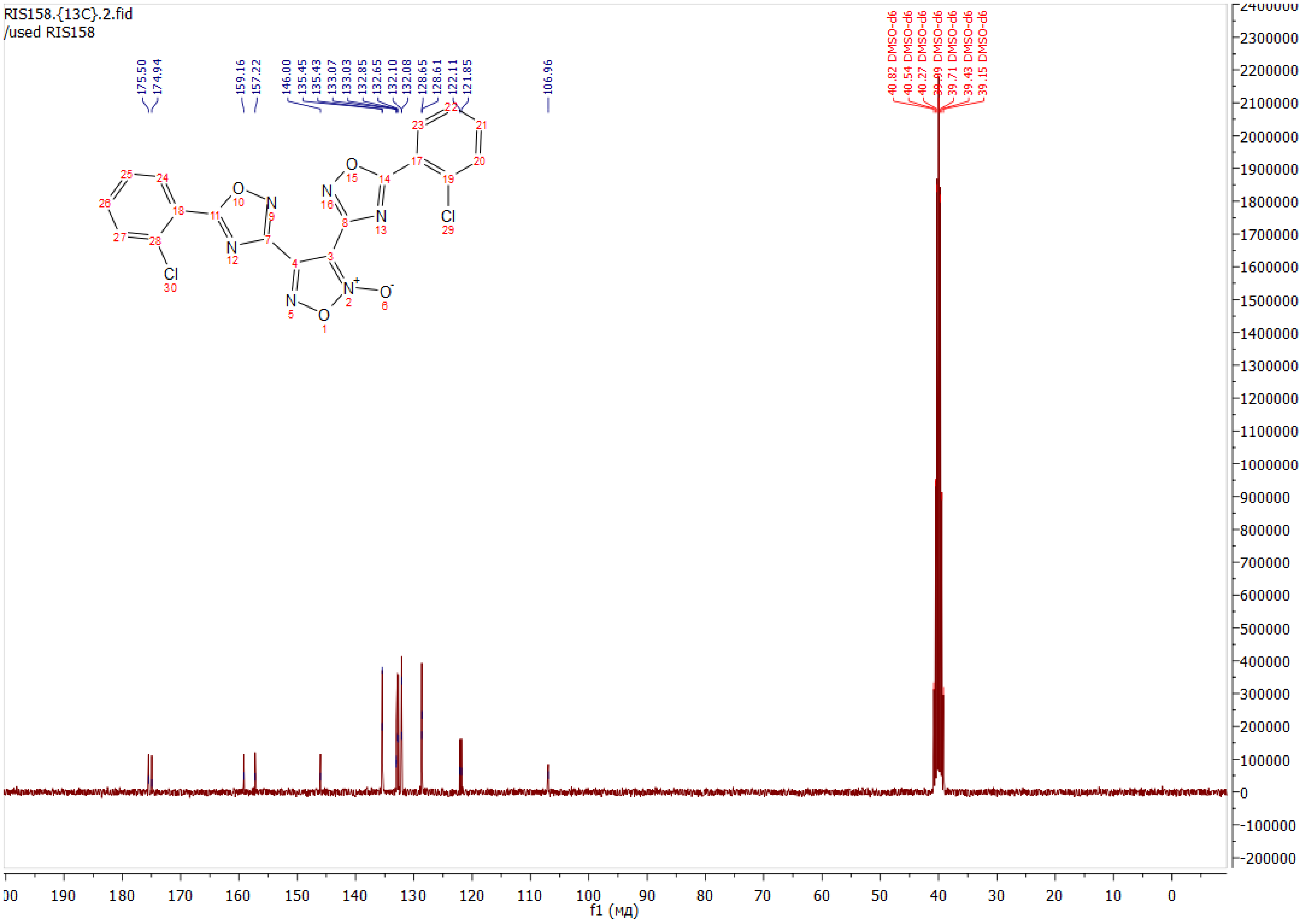

Figure S22. ^{13}C NMR spectrum of **2k**, DMSO-[d_6]

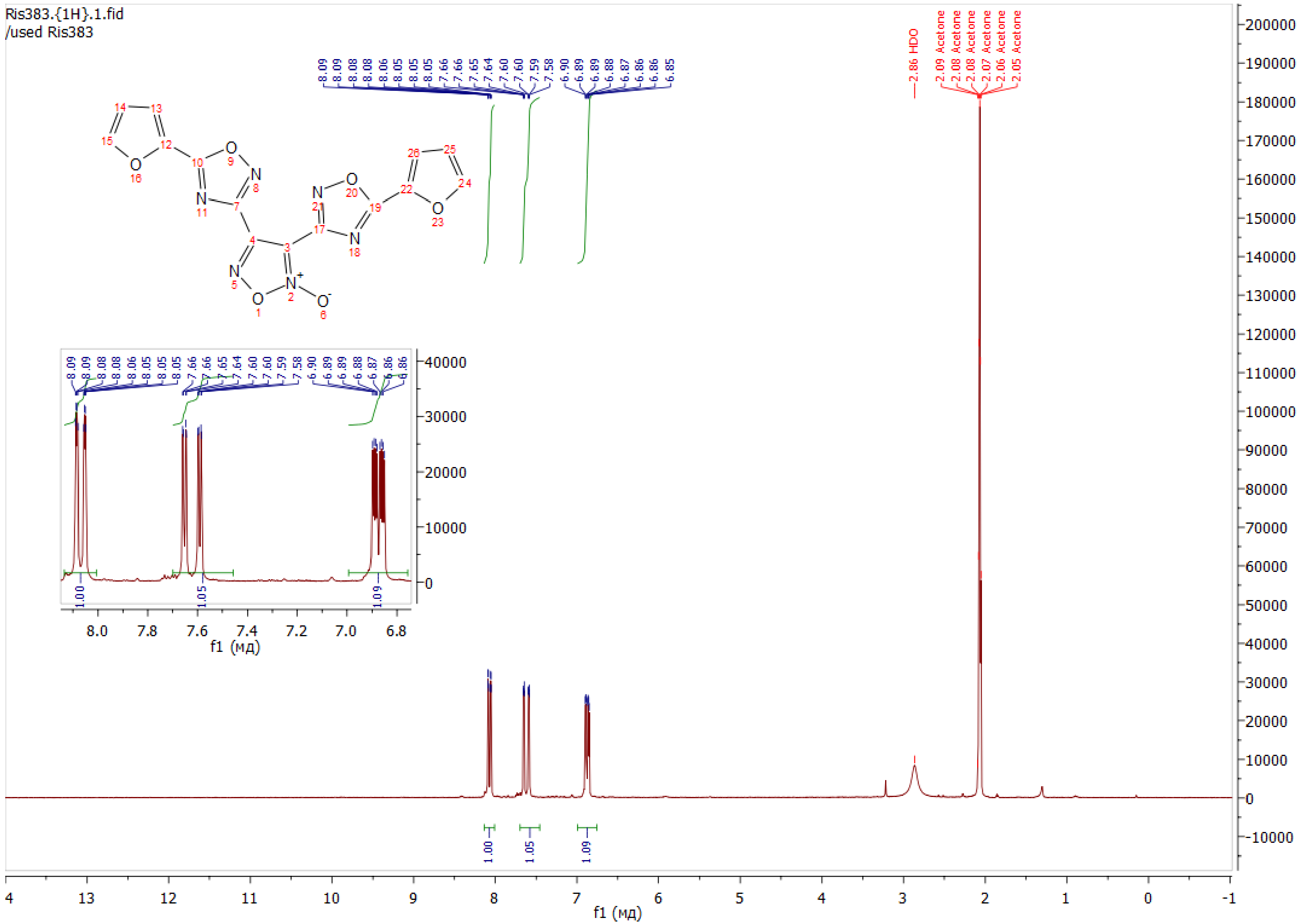

Figure S23. ^{1}H NMR spectrum of **2l**, Acetone-[d$_6$]

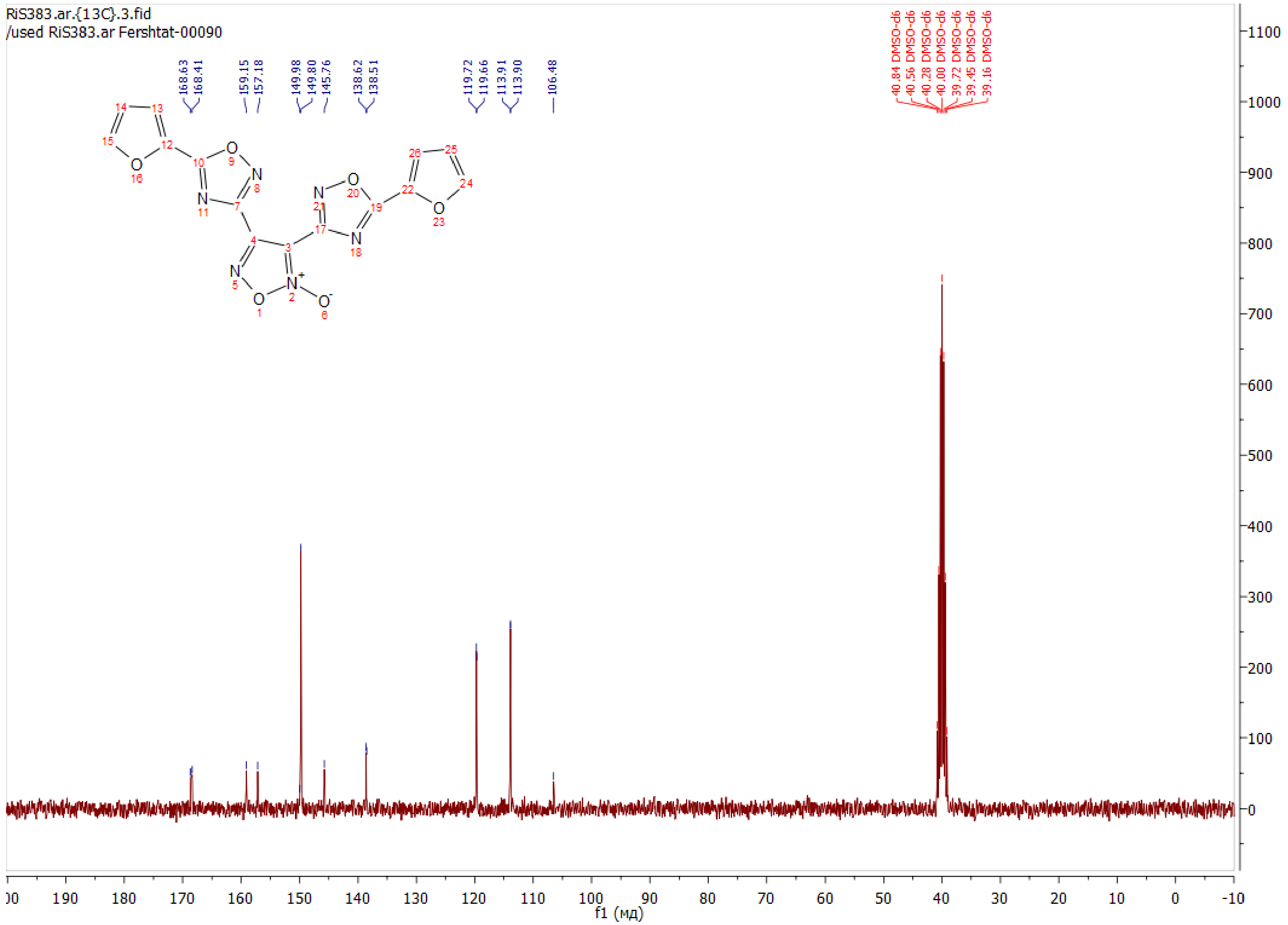

Figure S24. ^{13}C NMR spectrum of **2l**, Acetone-[d$_6$]

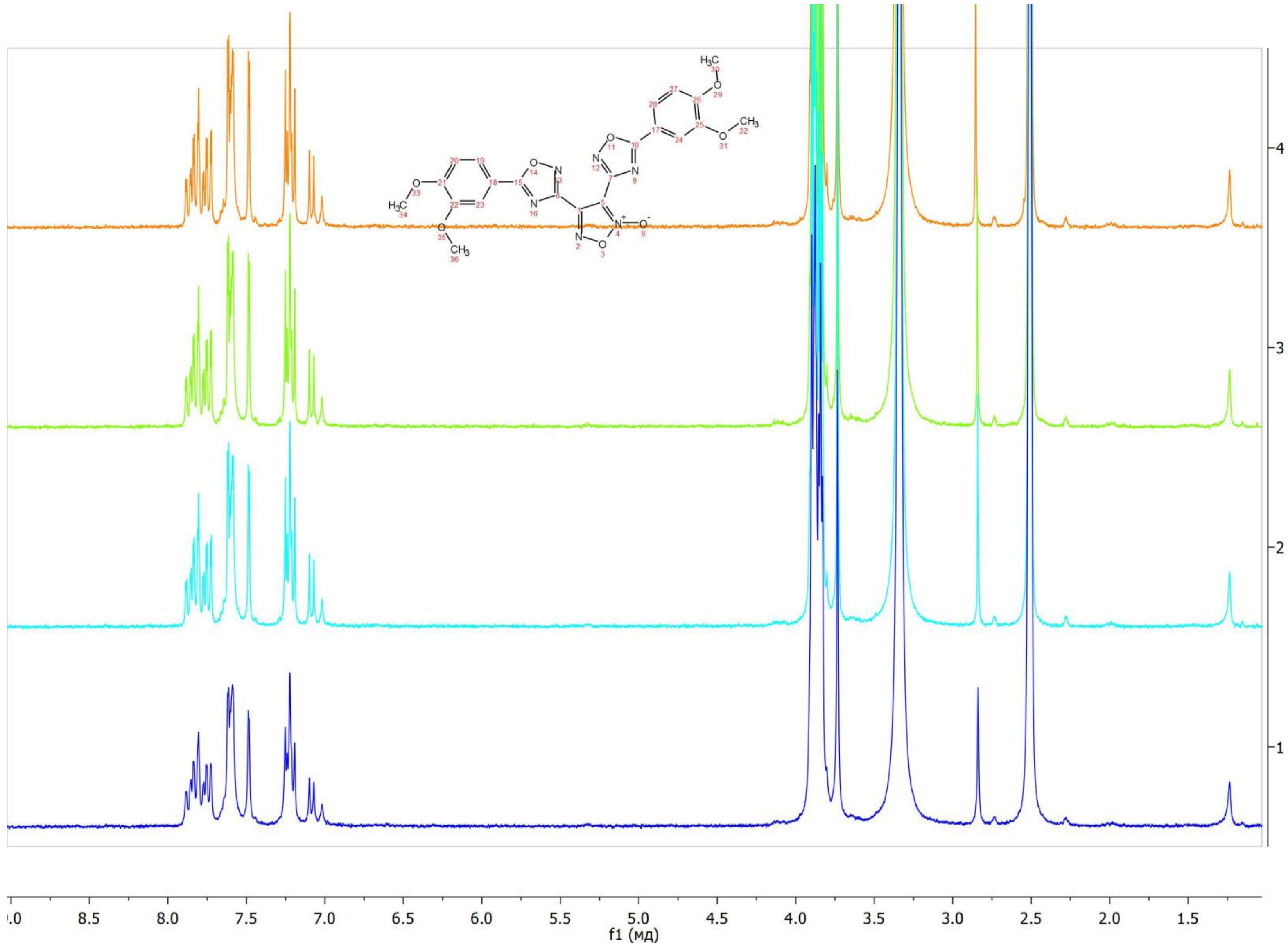

Figure S25. Stability of the representative compound **2j** for 1 h, 20 h, 48 h and 120 h in DMSO-[d_6] according to ^1H NMR spectroscopy.

S2. Crystallographic data

X-ray diffraction study of **2f** was performed using a Bruker Quest diffractometer (MoKa-radiation, graphite monochromator, ω-scans) equipped with a Photon-II area-detector at the facilities of the JRC PMR IGIC RAS. The **2f** crystals are twins; the corresponding diffraction data were indexing using the least-squares search implemented in the CELL_NOW routine and further validation of reciprocal space. The intensity data were integrated by the SAINT program [1] and were corrected for absorption and decay using SADABS [2]. Both structures were solved by dual space methods using SHELXT [3] and refined on F^2 using SHELXL-2018 [4]. Positions of hydrogen atoms were found from difference Fourier synthesis and then refined invoking isotropic approximation. All non-hydrogen atoms were refined with individual anisotropic displacement parameters. The main crystallography data and refinement parameters are given in the Table S2.1. The CCDC number 2388377 contains all the additional information on crystal structure and the details of refinement. The full optimization of structures of isolated molecule of **2f** were performed using the Gaussian 09 program (rev. D01) [5] at the PBE0 [6] /def2TZVP level with the Grimme's D3 dispersion corrections and Becke-Jonson damping [7]. Tight convergence criteria were used for the optimization procedures. Equilibrium structures of compound correspond to minimums on potential energy surface according to the calculations of Hessian of electronic energy (ultrafine grids, no imaginary modes were found). The root mean squared difference between the optimized and crystal conformation was calculated with the positions of hydrogen atoms being optimized at the same level for crystal conformation.

Table S1. Main crystallography data and refinement details for the **2f** structure.

	**2f**
Formula	$C_{16}H_8N_8O_4$
Molecular mass	376.30
T, K	100
Crystal system	Monoclinic
Space group	$P2_1/c$
Z (Z')	4 (2)
a, Å	27.7162(6)
b, Å	7.1743(2)
c, Å	15.9018(4)
β, °	95.5310(10)
V, Å^3	3147.26(14)
d_{calc}, g·cm^{-3}	1.588
μ, cm^{-1}	1.21
F(000)	1536

$2\theta_{max}$, °	54
Number of reflections measured	16839
Independent reflections	6850
Reflections with I>2σ(I)	5806
Number of parameters	506
R1	0.0597
wR2	0.1652
GOF	1.035
Residual electron density, e·$Å^{-3}$(d_{min}/d_{max})	0.429/-0.308

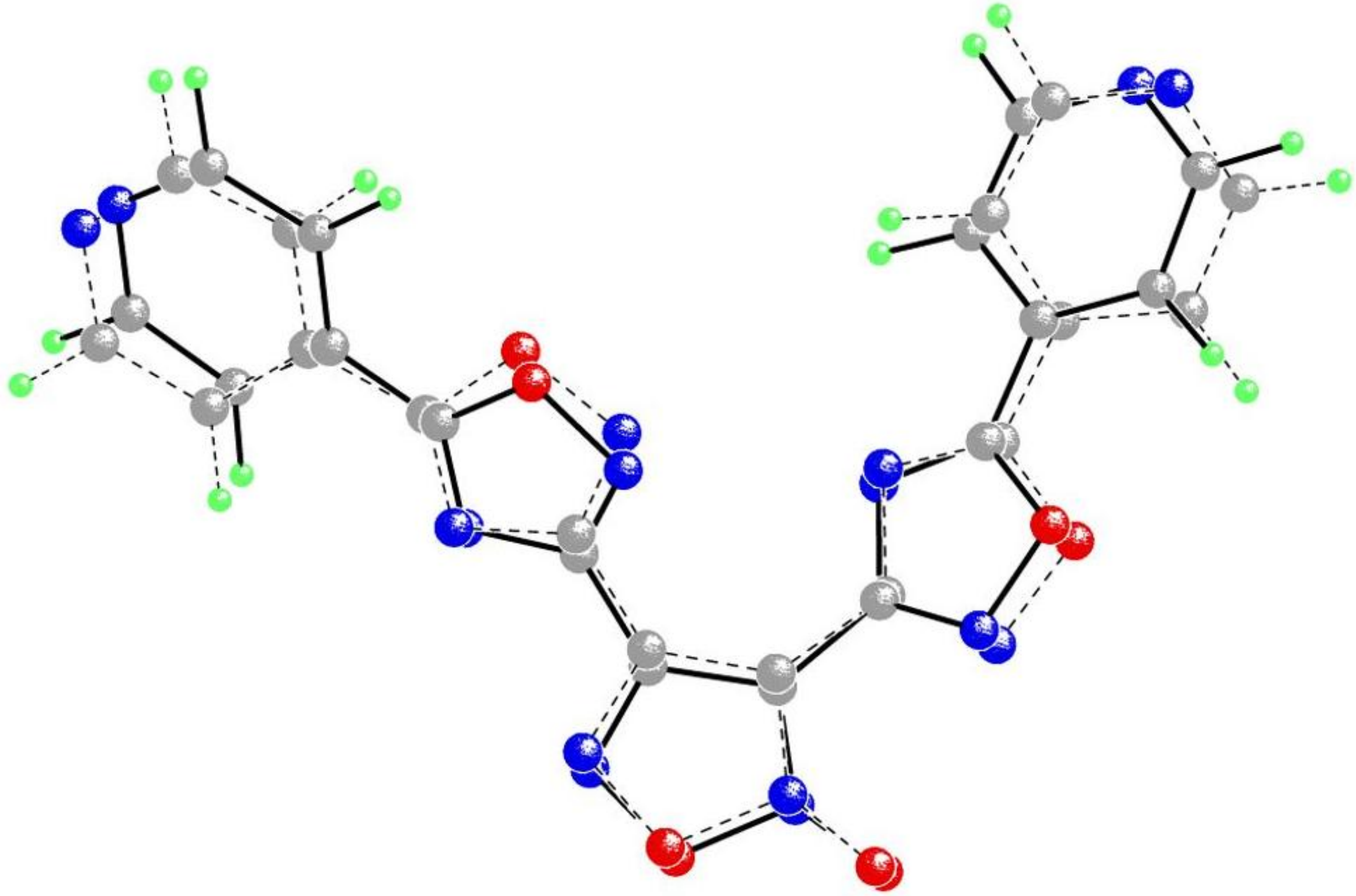

Figure S26. The best root mean square overlap of crystal (solid lines) and gas (dashed lines) conformations of **2f**.

S3. Biological activity

S3.1. Cell lines and culture conditions

AB1 (mouse malignant mesothelioma cell line) and JU77 (human mesothelioma cell line) were obtained from Cell Bank Australia; MRC-5 (human embryonic lung fibroblast cell line) was obtained from ATCC. AB1 and JU77 were cultured in RPMI containing 10% FBS and 1% penicillin–streptomycin (10 000 U mL^{-1}); MRC-5 was cultured in MEM containing 10% FBS, 1% penicillin–streptomycin (10 000 U mL^{-1}), and 1% L-glutamine (200 mM). Cells were grown in tissue culture flasks (75 cm^2 and 25 cm^2, SPL Life Sciences). All cell lines were grown at 37 °C in a humidified atmosphere containing 95% air and 5% CO_2. All drug stock solutions were prepared in DMSO, and the final concentration in the medium did not exceed 1%, at which cell viability was not inhibited.

S3.2. Evaluation of anticancer activity

The cytotoxicity of compounds was determined using the MTT colorimetric test. Cells were harvested from culture flasks by trypsinization and seeded into Cellstar 96-well microculture plates at a seeding density of 6000 cells per well (6×10^4 cells per mL). After the cells were allowed to resume exponential growth for 24 h, they were exposed to drugs at different concentrations in media for 72 h. Tested compounds were diluted in the complete medium at the desired concentrations added to each well (100 μL) and serially diluted to other wells. After exposure for 72 h, the medium was replaced with MTT (5 mg mL^{-1}, 100 μL per well) and incubated for an additional 50 min. Subsequently, the medium was aspirated, and the purple formazan crystals formed in viable cells were dissolved in DMSO (100 μL per well). Optical densities were measured at 570 nm using the BioTek Synergy H1 microplate reader. The quantity of viable cells was expressed in terms of treated/control (T/C) values by comparison to untreated control cells, and 50% inhibitory concentrations (IC_{50}) were calculated from concentration–effect curves by interpolation. The evaluation was based on means from at least three independent experiments, each comprising three replicates per concentration level.

S3.3. Evaluation of NO release *in vitro*

The NO release *in vitro* of compounds was determined using the Griess Reagent from Nitrite/Nitrate Assay colorimetric kit (Sigma-Aldrich, Germany). Cells were harvested from culture flasks by trypsinization and seeded into Cellstar 96-well microculture plates at a seeding density of 10,000 cells per well (1×10^5 cells per mL). After the cells were allowed to resume exponential growth for 24 h, they were exposed to 100 μL drugs at a concentration of 25 μM in colorless RPMI media (Gibco, UK) for 24 h. After exposure for 24 h, 10 μL of Griess Reagent A was added to each well and the plate was mixed using a horizontal shaker at room temperature for 5 min. Then 10 μL of Griess Reagent B was added to each well and the plate was mixed using a horizontal shaker at room temperature for 15 min. NO release presented as a fold change normalized to untreated control (no drug), while $NaNO_2$ Standard Solution from the kit at concentrations 0, 2, 4, and 8 nM (100 μL per well) was prepared as the positive control for the standard curve for each experiment. The

evaluation was based on means from at least three independent experiments, each comprising three replicates per concentration level.

S3.4. JC1 assay

AB1 cells were seeded into Cellstar 6-well plates (Greiner Bio-One) at a density of 3 × 10^5 cells/well (2 mL per well) and were allowed to resume exponential growth for 24 hours. Subsequently, the medium was removed, and the cells were washed with PBS (2 × 1 mL). Afterwards, the cells were incubated with the compounds of interest 25 μM concentrations for 24 hours at 37°C and 5% CO_2. For the positive control, cells were pre-treated with CCCP (20 μM) for 30 minutes prior to JC-1 staining. Following the completion of treatment, the culture supernatants were carefully collected. The adherent cells were then harvested by trypsinization using 200 μL of trypsin per well, with a 5-minute incubation period at 37°C and 5% CO_2 to ensure complete detachment. After trypsinization, the cell suspension was centrifuged at 3 × 10^3 rpm for 5 minutes to pellet the cells. The resulting cell pellets were gently resuspended in 1 mL of JC-1 solution (2 μM in pre-warmed PBS) for 30 minutes at 37°C and 5% CO_2, protected from light to prevent dye degradation. After the incubation, the cells were washed with PBS (2 × 1 mL) by centrifugation at 3 × 10^3 rpm for 5 minutes. Finally, the cells were resuspended in 500 μL of ice-cold PBS for flow cytometry analysis. Flow cytometry was performed using a CytoFLEX Flow Cytometer (Beckman Coulter, Brea, CA, USA) equipped with a 488 nm laser for excitation and two emission channels (FL1 channel for JC-1 monomers and FL2 channel for JC-1 aggregates). The resulting dot plots were acquired from 20,000 events and quantified using FlowJo software (version 10.8.0, BD Biosciences, San Jose, CA, USA).

S3.5. ROS detection

AB1 cells were seeded into the confocal dish (Biosharp) at a density of 5 × 10^5 cells/well (1 mL per well). The cells were allowed to resume exponential growth for 24 h. The cell culture medium was aspirated and washed with PBS (2 × 1 ml). In a dimly lit setting, a solution of H_2DCFDA (2',7'-dichlorodihydrofluorescein diacetate) in 1× HBSS (10 μM, 1 mL) was added to each well and allowed to incubate for 5 min at 37 °C. Following this, the H_2DCFDA solution was aspirated, and the wells were rinsed with HBSS (2 × 1 ml). Subsequently, the cells were treated with fresh colourless cell culture medium containing compounds of interest at their respective 100 μM concentrations for 2 h at 37 °C. Following the incubation period, the drug-containing medium was removed, and the wells were washed with PBS (2 × 1 ml). Subsequently, the cells were treated with a culture medium containing 1 μM of Mitotracker TM Deep Red, 1 μM ER-Tracker™ Blue-White DPX and 1 μg/mL of Hoechst 33342 for 30 minutes. Following this incubation, the cells were washed with PBS (2 × 1 ml). The samples were then protected from light with aluminium foil before imaging. Images were captured using Laser Confocal Scanning Microscope (Leica SPE) and analyzed using the Microscope Software Platform LAS X Life Science.

S3.6. Annexin V/PI assay

AB1 cells were seeded into Cellstar 6-well plates (Greiner Bio-One) at a density of 5×10^5 cells/well (1 ml per well). The cells were allowed to resume exponential growth for 24 h. The medium was removed and cells were washed with PBS (2 × 1 mL). Afterwards the cells were incubated with a fresh medium containing compounds of interest 25 µM concentrations for 24 h at 37 °C. After the treatment, the supernatant was collected in 2 mL microtubes, centrifuged (300 × g, 5 min) and the media was removed. The adherent cells were then trypsinized with 400 µL of trypsin at 37 °C for 5 min. Next, 800 µL of medium was added to neutralize trypsin and the resulting cell suspension was combined with the supernatant. The wells were washed with 500 µL of PBS, which was also combined with the supernatant. The combined solutions were centrifuged (300 × g, 5 min), and the resulting pellets were washed with PBS (2 × 1 mL) and resuspended in 400 µL of Annexin V binding buffer. The cells were stained with Annexin V-FITC for 15 min in ice protected from light, and then propidium iodide (PI) was added just before each measurement. Cell death was evaluated by flow cytometry using a CytoFLEX Flow Cytometer equipped with a 488 nm laser and a 638 nm laser (Beckman Coulter, Brea, CA, USA). The cells were excited by the 488 nm laser, and fluorescence emissions were detected at 525/40 nm for FITC (Annexin V) and at 610/20 nm for ECD (PI). The resulting dot blots were acquired from 10,000 events and quantified using Flowjo software (version 10.8.0, BD Biosciences, San Jose, CA, USA). The percentage of apoptotic cells was determined by calculating the sum of cells in Q2 and Q3.

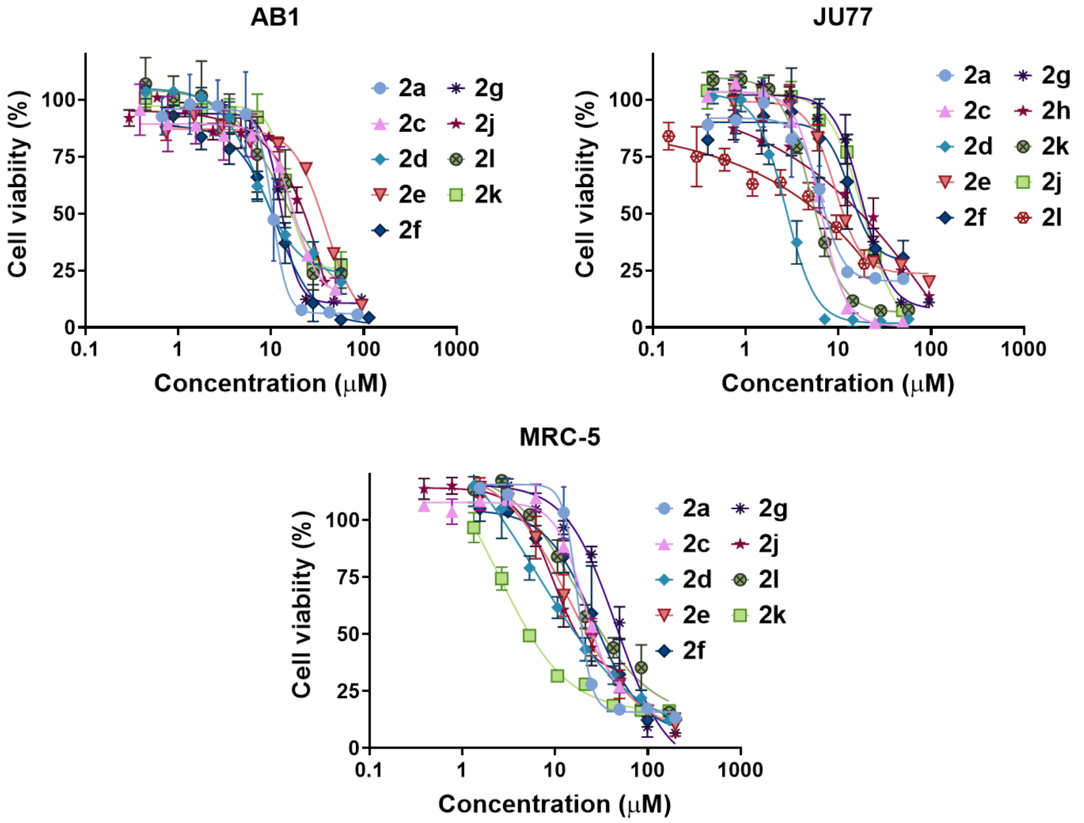

Figure S27. Concentration-effect curves for **2a-l** and **3a-l** in AB1, JU77, and MRC-5 cell lines upon 72 h exposure.

S4. References

1. Bruker. APEX-III. *Bruker AXS Inc.*, Madison, Wisconsin, USA, **2018**.

2. L. Krause, R. Herbst-Irmer, G. M. Sheldrick, D. Stalke, Comparison of silver and molybdenium microfocus X-ray sources for single-crystal structure determination. *J. Appl. Cryst.*, **2015**, *48*, 3-10.

3. G. M. Sheldrick, SHELXT – Integrated space-group and crystal structure determination. *Acta Crystallogr. Sect. A*, **2015**, *71*, 3-8.

4. G. M. Sheldrick, Crystal structure refinement with SHELXL. *Acta Crystallogr. Sect C*, **2015**, *71*, 3-8.

5. Gaussian 09, Revision D.01, M. J. Frisch, G. W. Trucks, H. B. Schlegel, G. E. Scuseria, M. A. Robb, J. R. Cheeseman, G. Scalmani, V. Barone, G. A. Petersson, H. Nakatsuji, X. Li, M. Caricato, A. Marenich, J. Bloino, B. G. Janesko, R. Gomperts, B. Mennucci, H. P. Hratchian, J. V. Ortiz, A. F. Izmaylov, J. L. Sonnenberg, D. Williams-Young, F. Ding, F. Lipparini, F. Egidi, J. Goings, B. Peng, A. Petrone, T. Henderson, D. Ranasinghe, V. G. Zakrzewski, J. Gao, N. Rega, G. Zheng, W. Liang, M. Hada, M. Ehara, K. Toyota, R. Fukuda, J. Hasegawa, M. Ishida, T. Nakajima, Y. Honda, O. Kitao, H. Nakai, T. Vreven, K. Throssell, J. A. Montgomery, Jr., J. E. Peralta, F. Ogliaro, M. Bearpark, J. J. Heyd, E. Brothers, K. N. Kudin, V. N. Staroverov, T. Keith, R. Kobayashi, J. Normand, K. Raghavachari, A. Rendell, J. C. Burant, S. S. Iyengar, J. Tomasi, M. Cossi, J. M. Millam, M. Klene, C. Adamo, R. Cammi, J. W. Ochterski, R. L. Martin, K. Morokuma, O. Farkas, J. B. Foresman, D. J. Fox, Gaussian, Inc., Wallingford CT, **2016**.

6. J. Perdew, M. Ernzerhof and K. Burke, Rationale for mixing exact exchange with density functional approximations. *J. Chem. Phys.*, **1996**, *105*, 9982-9985; C. Adamo and V. Barone, Toward reliable density functional methods without adjustable parameters: The PBE0 model. *J. Chem. Phys.*, **1999**, *110*, 6158-6170.

7. S. Grimme, S. Ehrlich and L. Goerigk, Effect of the damping function in dispersion corrected density functional theory. *J. Comput. Chem.*, **2011**, *32*, 1456-1465.
